# Crystal structures of five gold(I) complexes with methyl­piperidine ligands

**DOI:** 10.1107/S2056989023010940

**Published:** 2023-01-19

**Authors:** Cindy Döring, Peter G. Jones

**Affiliations:** a Institut für Anorganische und Analytische Chemie, Technische Universität Braunschweig, Hagenring 30, D-38106 Braunschweig, Germany; Universität Greifswald, Germany

**Keywords:** crystal structure, gold, methyl­piperidine, hydrogen bonds, aurophilic inter­actions.

## Abstract

In bis­(4-methyl­piperidine-κ*N*)gold(I) chloride, [Au(C_6_H_13_N)_2_]Cl (**1**), the methyl groups are, as expected, equatorial at the piperidine ring, but the Au atom is axial; this is the case for all five structures reported here, as is the expected linear coordination at the Au atom. Hydrogen bonding of the form N—H⋯Cl^−^⋯H—N leads to inversion-symmetric dimers, which are further connected by C—H⋯Au contacts. Bis(4-methyl­piperidine-κ*N*)gold(I) di­chlorido­aurate(I), [Au(C_6_H_13_N)_2_][AuCl_2_] (**2**), also forms inversion-symmetric dimers; these involve aurophilic inter­actions and three-centre hydrogen bonds of the form NH(⋯Cl)_2_. Bis(4-methyl­piperidine-κ*N*)gold(I) di­bromido­aurate(I), [Au(C_6_H_13_N)_2_][AuBr_2_] (**3**), is isotypic to **2**. The 1:1 adduct chlorido­(4-methyl­piperidine-κ*N*)gold(I) bis­(4-methyl­piperidine-κ*N*)gold(I) chloride, [Au(C_6_H_13_N)_2_]Cl·[AuCl(C_6_H_13_N)] (**4**), crystallizes as its di­chloro­methane solvate. The asymmetric unit contains two formula units, in each of which the chloride anion accepts a hydrogen bond from the cation and from the neutral mol­ecule, and the two Au atoms are linked *via* an aurophilic inter­action. A further hydrogen bond leads to inversion-symmetric dimers. The asymmetric unit of bis­(2-methyl­piperidine-κ*N*)gold(I) chloride, [Au(C_6_H_13_N)_2_]Cl (**5**), contains two ‘half’ cations, in which the Au atoms lie on twofold axes, and a chloride ion on a general position. Within each cation, the relative configurations at the atoms N and C2 (which bears the methyl substituent) are *R*,*S*. The twofold-symmetric dimer involves two N—H⋯Cl^−^⋯H—N units and an aurophilic contact between the two Au atoms.

## Abstract 2

Five structures with methyl­piperidine ligands and gold(I) centres all exhibit linear geometry at the Au atom, and equatorial and axial positions, respectively, for the methyl groups and Au atoms at the piperidine rings. The packing involves hydrogen bonding and aurophilic inter­actions.

## 1. Chemical context

We have published structures of several amine complexes of gold halides and pseudohalides, many of which can be obtained in crystalline form despite the apparent unsuitability of complexes involving a hard donor atom (nitro­gen) and a soft metal centre (gold). Some, however, are only stable in the presence of excess ligand. The structures often involve aurophilic inter­actions (for Au^I^ complexes; reviewed by Schmidbaur & Schier, 2008 ▸, 2012 ▸), hydrogen bonding [see *e.g.* Brammer (2003 ▸) for a description of hydrogen bonding to metal-bonded halogens], gold–halogen contacts or halogen–halogen contacts (see *e.g.* Metrangelo, 2008 ▸). Extensive background material, including a summary of product types, can be found in Part 12 of this series (Döring & Jones, 2023*a* ▸), which presented complexes involving piperidine and pyrrolidine complexes, and further relevant literature is cited in Part 13 (Döring & Jones, 2023*b* ▸), which dealt with the isotypic complexes bis­(morpholine)­gold(I) chloride and bis­(morph­o­line)­gold(I) bromide. The current paper extends these studies to complexes of gold(I) with the ligands 4-methyl­piperidine and 2-methyl­piperidine (abbreviated henceforth as 4-Me-pip and 2-Me-pip): bis­(4-methyl­piperidine)­gold(I) chloride, [Au(4-Me-pip)_2_]Cl, **1**; bis­(4-methyl­piperidine)­gold(I) di­chlorido­aurate(I), [Au(4-Me-pip)_2_][AuCl_2_], **2**; bis­(4-methyl­piperidine)­gold(I) di­bromido­aurate(I), [Au(4-Me-pip)_2_] [AuBr_2_], **3** (isotypic to **2**); the adduct chlorido­(4-methyl­piperidine)­gold(I) bis­(4-methyl­piperidine)­gold(I) chloride, [AuCl(4-Me-pip)]·[Au(4-Me-pip)_2_]Cl, as its di­chloro­methane solvate **4**; and bis­(2-methyl­piperidine)­gold(I) chloride, [Au(2-Me-pip)_2_]Cl, **5**. 
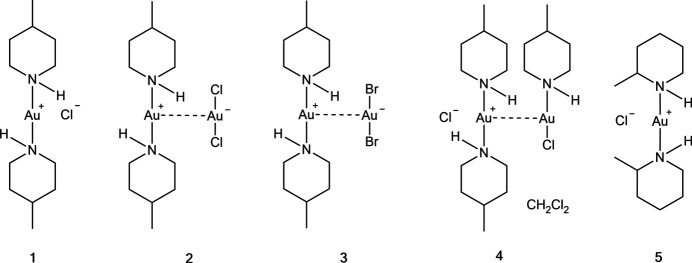


## 2. Structural commentary

At the outset we comment, as usual: for structures that contain more than one residue in the asymmetric unit, the distinction between the categories *Structural commentary* (which generally refers to the asymmetric unit) and *Supra­molecular features* becomes blurred, especially when atoms occupy special positions (as for compound **5** here).

Selected mol­ecular dimensions are presented in Tables 1 ▸–5 ▸ ▸ ▸ ▸, with hydrogen bonds in Tables 6 ▸–10 ▸ ▸ ▸ ▸.

**Figure 1:**
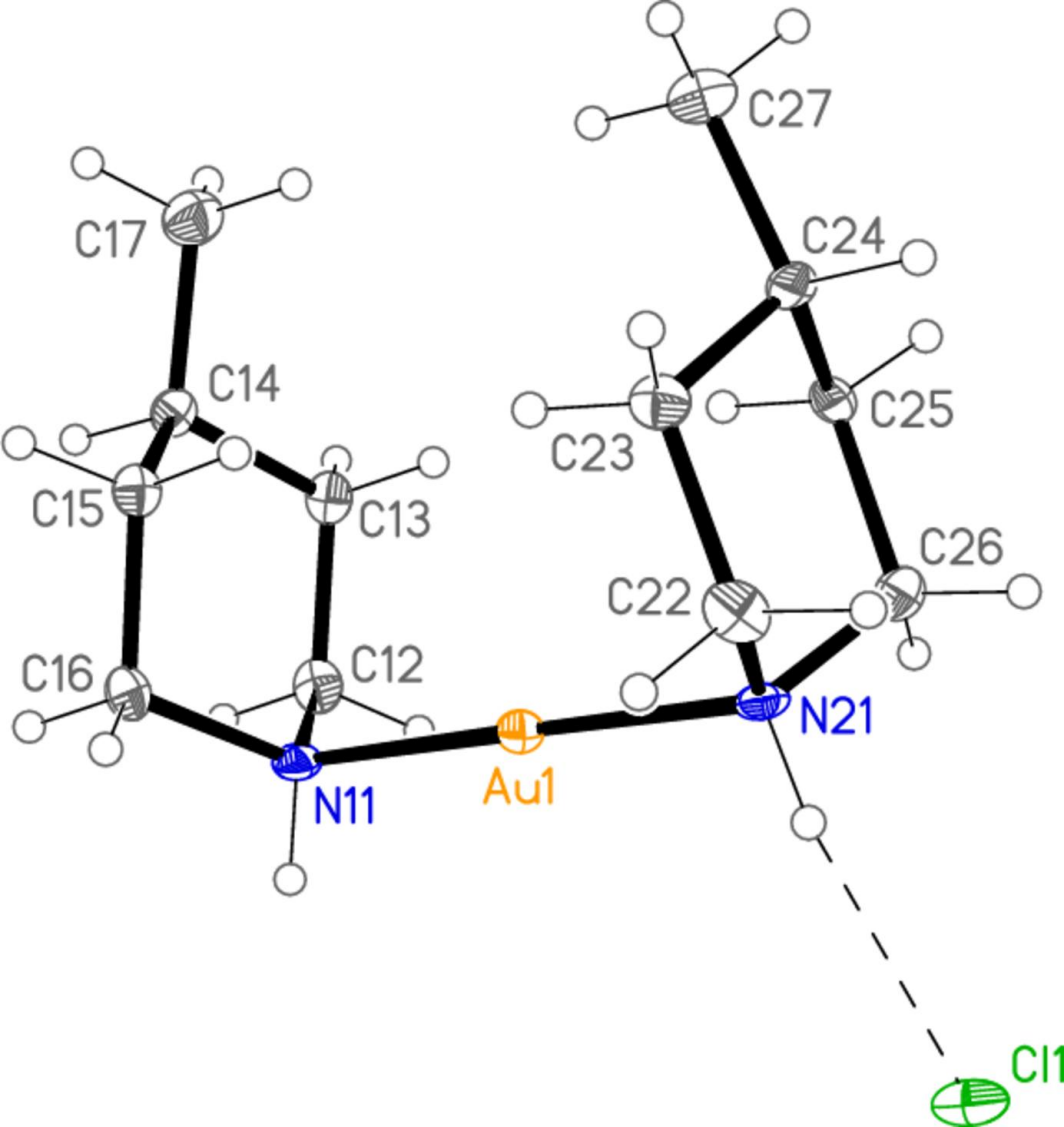
The asymmetric unit of compound **1** in the crystal, with ellipsoids at the 50% probability level. The dashed line represents a hydrogen bond.

Fig. 1 ▸ shows the asymmetric unit of compound **1**, which consists of one formula unit [Au(4-Me-pip)_2_]Cl and contains one classical hydrogen bond N21—H02⋯Cl1 (Table 6 ▸). All atoms lie on general positions. Selected geometric parameters are presented in Table 1 ▸. The geometry at the Au atom is, as expected, linear (as is the case for all structures presented in this paper). The substituents of the piperidine ring occupy different position types; whereas the methyl groups are equatorial, which would be expected, the Au atoms are axial (*cf.* absolute C_meth­yl_—C—C—C and Au—N—C—C torsion angles of approximately 180 or 60° respectively in Table 1 ▸). The axial configuration of Au atoms with respect to piperidine ligands has been noted in our previous papers (Döring & Jones, 2023*a* ▸,*b* ▸), although it is not always observed; in the AuCl(piperidine) tetra­mer (Guy *et al.*, 1977 ▸), for instance, the Au atoms lie equatorially with respect to the ring. The piperidine rings eclipse each other when viewed along the direction N11⋯N21, with pseudo torsion angles C26—N21⋯N11—C12 = 2.9 (3)° and C22—N21⋯N11—C16 = 2.2 (3)°. Because of the contrast with compound **5** (see below), we comment here that the 4-Me-pip complexes **1**–**4** are achiral, because of the local mirror planes through the atoms N, C-4, C_meth­yl_ and Au.

**Figure 2:**
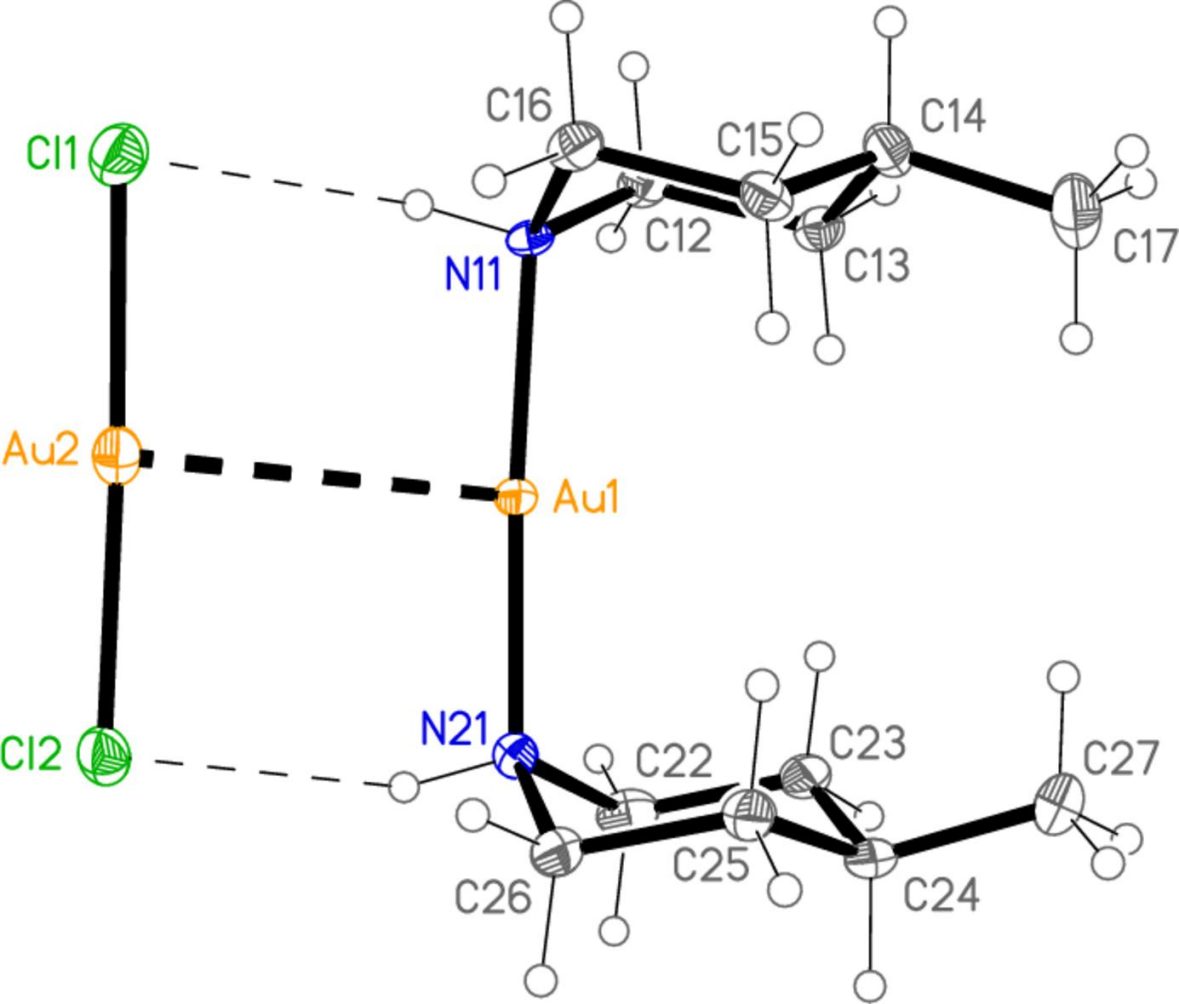
The asymmetric unit of compound **2** in the crystal, with ellipsoids at the 50% probability level. The dashed lines represent hydrogen bonds (thin) or the aurophilic inter­action (thick).

**Figure 3:**
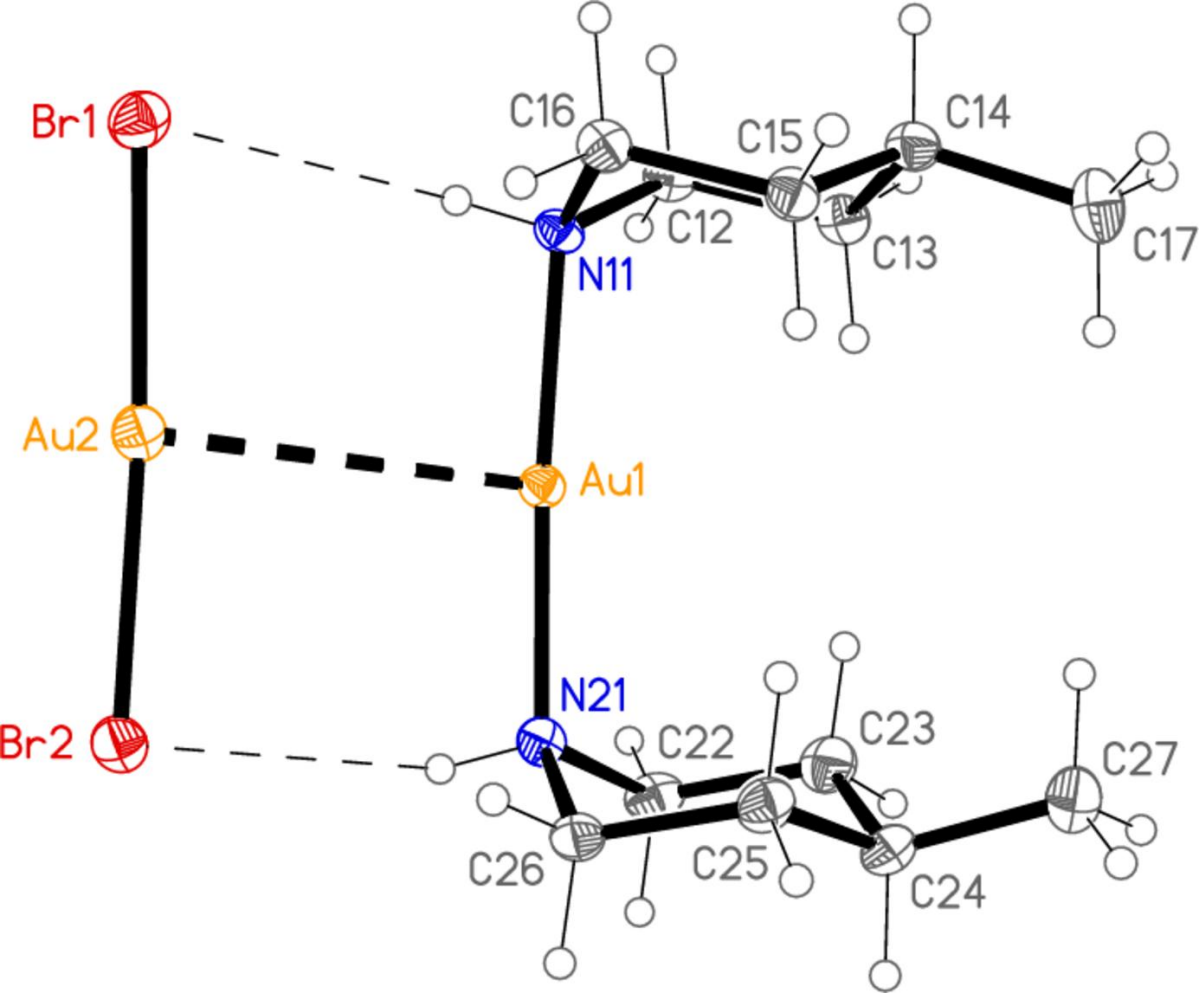
The asymmetric unit of compound **3** in the crystal, with ellipsoids at the 50% probability level. The dashed lines represent hydrogen bonds (thin) or the aurophilic inter­action (thick).

The structure of compound **2** is shown in Fig. 2 ▸; the asymmetric unit contains one [Au(4-Me-pip)_2_]^+^ cation and one [AuCl_2_]^−^ anion, a composition corresponding to type III in our arbitrary classification of products (Döring & Jones, 2023*a* ▸), and already observed during our studies of secondary amine complexes (Döring & Jones, 2018 ▸) for [Au(Et_2_NH)_2_] [AuBr_2_]. All atoms lie on general positions. Selected geometric parameters are presented in Table 2 ▸. The configurations of the methyl group and the gold substituent at the piperidine ring are the same as for **1**, namely equatorial and axial, respectively. The anion and cation are connected by two N—H⋯Cl hydrogen bonds (Table 7 ▸) and the aurophilic contact Au1⋯Au2, and the coordination axes are thus almost parallel, with torsion angles of *ca* 0 and 180° around Au1⋯Au2 [*e.g*. N11—Au1⋯Au2—Cl1 = −3.1 (2)°, N11—Au1⋯Au2—Cl2 = −177.5 (2)°]. As in **1**, the piperidine rings eclipse each other when viewed along the direction N11⋯N21, with pseudo torsion angles C12—N11⋯N21—C22 = 2.1 (3)° and C11—N11⋯N21—C26 = 1.0 (3)°. Compound **3** is isotypic to **2**; it is shown in Fig. 3 ▸, with mol­ecular dimensions and hydrogen bond details in Tables 3 ▸ and 8 ▸, but is not discussed further.

**Figure 4:**
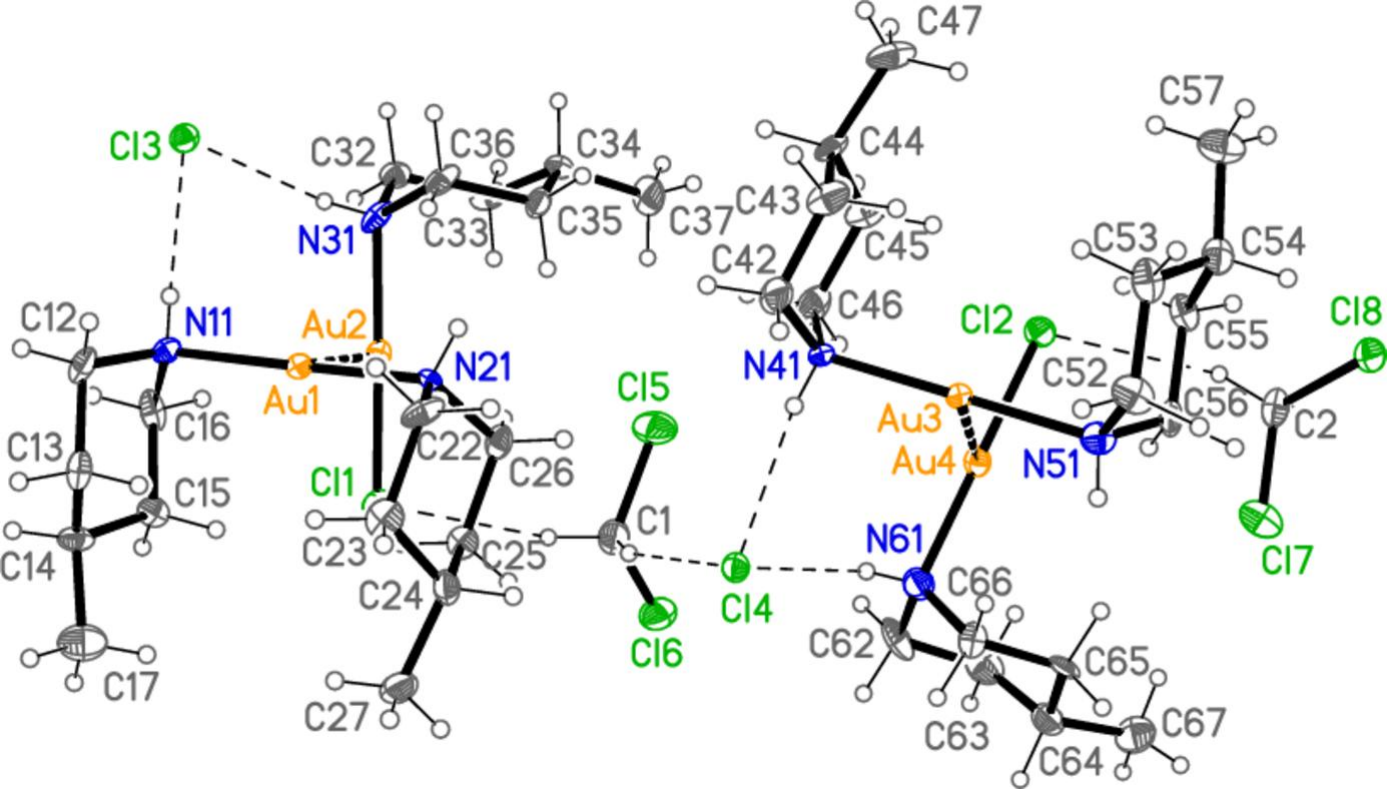
The asymmetric unit of compound **4** in the crystal, with ellipsoids at the 50% probability level. The dashed lines represent hydrogen bonds (thin) or aurophilic contacts (thick).

Compound **4** is a 1:1:1 mixture of the mol­ecular complex [AuCl(4-Me-pip)], the ionic [Au(4-Me-pip)_2_]Cl (thus corresponding to a mixture of types I and II, as established for the corresponding pyrrolidine derivative; Döring & Jones, 2023*a* ▸) and di­chloro­methane. The di­chloro­methane is well-ordered. All atoms lie on general positions. The asymmetric unit consists of two closely similar formula units and is shown in Fig. 4 ▸. Selected geometric parameters are presented in Table 4 ▸. As usual in this series of compounds, all Au atoms occupy an axial position at the piperidine rings, and all methyl groups are equatorial. In each formula unit, the chloride anion accepts one hydrogen bond (Table 9 ▸) each from the cation and the neutral mol­ecule, and the two Au atoms are linked *via* an aurophilic inter­action. The H⋯Cl^−^⋯H angles are 83 (3)° at Cl3 and 80 (3)° at Cl4. The coordination axes at the linked Au atoms are approximately perpendicular to each other, with torsion angles *e.g.* N11—Au1⋯Au2—N31 = 85.3 (3)°, N11—Au1⋯Au2—Cl1 = −93.2 (2)° for the first formula unit and N41—Au3⋯Au4—N61 = −84.6 (3)°, N41—Au3⋯Au4—Cl2 = 95.0 (2)° for the second. The di­chloro­methane mol­ecules form short C—H⋯Cl hydrogen bonds to the chlorido ligands of the same formula unit and to the chloride anion of the other formula unit (for C1—H1*A*⋯Cl4 within the asymmetric unit but for C2—H2*B*⋯Cl3 *via* a glide plane, see section 3). The short contacts H16*A*⋯Au2 and H46*B*⋯Au4 (Table 9 ▸) might be regarded as forced by the formation of the hydrogen-bonded dimer (see Section 3).

**Figure 5:**
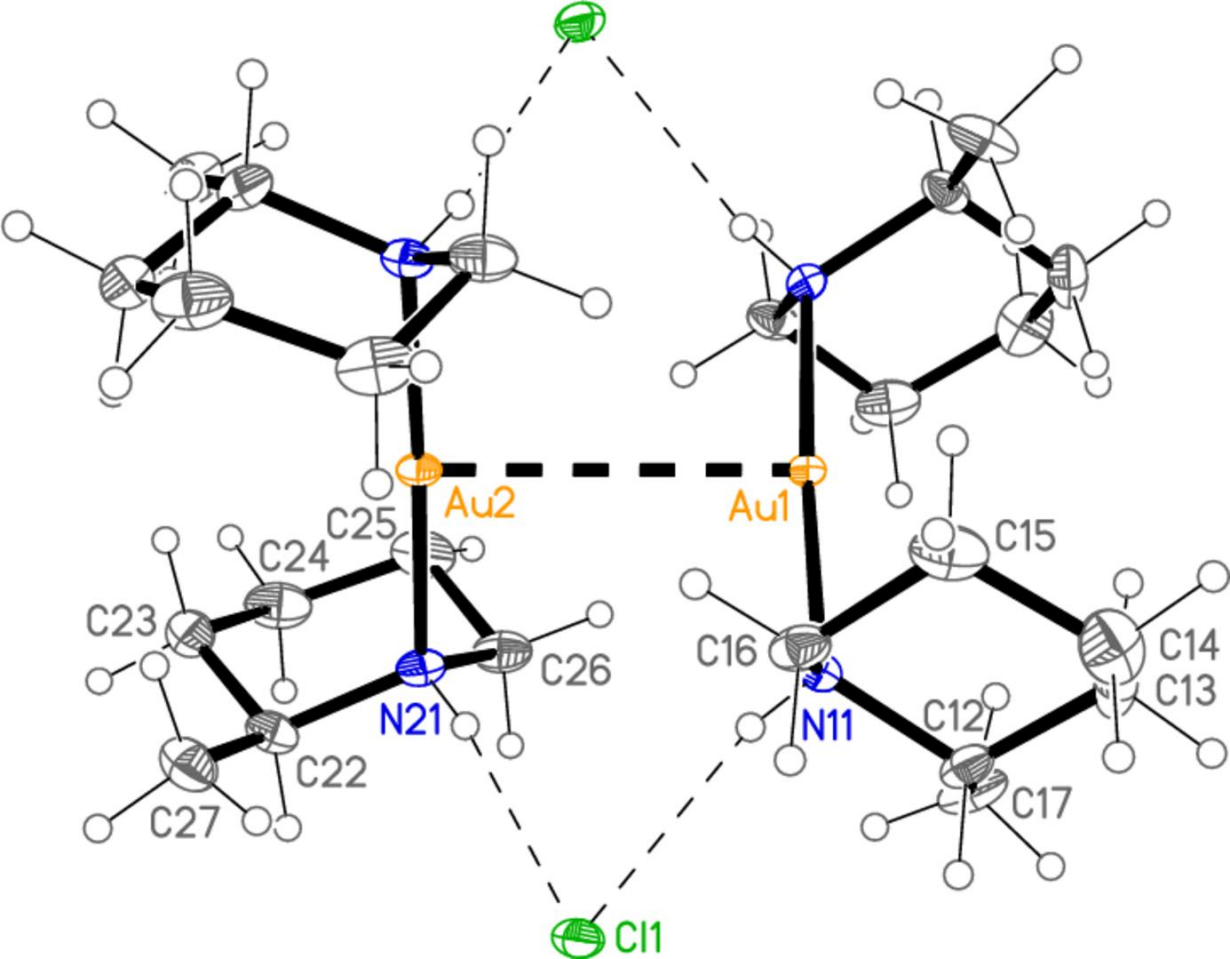
The hydrogen-bonded dimer of compound **5** in the crystal, with ellipsoids at the 30% probability level. The dashed lines represent hydrogen bonds (thin) or an aurophilic contact (thick).

Compound **5** is the only complex of 2-Me-pip for which a structure was obtained. Selected geometric parameters are presented in Table 5 ▸. The asymmetric unit contains two [Au(2-Me-pip)_2_]^+^ cations, for both of which the Au atoms lie on the twofold axis 0.5, *y*, 0.25, and one chloride ion on a general position. Fig. 5 ▸ shows the twofold-symmetric dimer, which involves two N—H⋯Cl^−^⋯H—N units [H—Cl^−^⋯H = 75.4 (16)°] and an aurophilic contact between the two Au atoms. Again, both Au atoms occupy an axial position at the piperidine rings, and both methyl groups are equatorial. The presence of two stereocentres in each piperidine ring, at the nitro­gen and the methyl-substituted carbon atom, means that various diastereomers of the cation of **5** are formally possible, but their number is limited (i) by the preferences of gold for an axial and of the methyl group for an equatorial position, leading to configurations of *R*,*S* at N11 and C12 respectively, and (ii) by the twofold axis through the Au atom, so that the second piperidine of each cation is also *R*,*S* (it is conceivable that a different form of **5** might be obtained in which the two ligands of the cation have opposite configurations, for instance if the Au atom lay on an inversion centre). The same relative configurations would apply to any 2-methyl­piperidine complex with an axially positioned metal, whereas an equatorially placed metal would lead to the same configuration for both centres (see *Database Survey* below). Of course, in the centrosymmetric space group *C*2/*c* the overall composition of **5** is a racemate. The coordination axes are inclined to each other at an angle of *ca* 64° [*cf*. torsion angle N11—Au1⋯Au2—N21 = −64.21 (14)°]. The central hydrogen-bonded ring has graph set 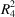 (12) (Bernstein *et al.*, 1995 ▸). The piperidine rings at each Au atom are mutually rotated, as viewed along the direction N⋯N′, but to a different extent [*cf*. pseudo torsion angles C12—N11⋯N11′—C12′ = −51.28 (5), C22—N21⋯N21′—C21′ = −15.23 (6)°; the primes indicate atom positions generated by the twofold axis].

## 3. Supra­molecular features

Supra­molecular features within the asymmetric units have already been discussed in the *Structural commentary* section.

**Figure 6:**
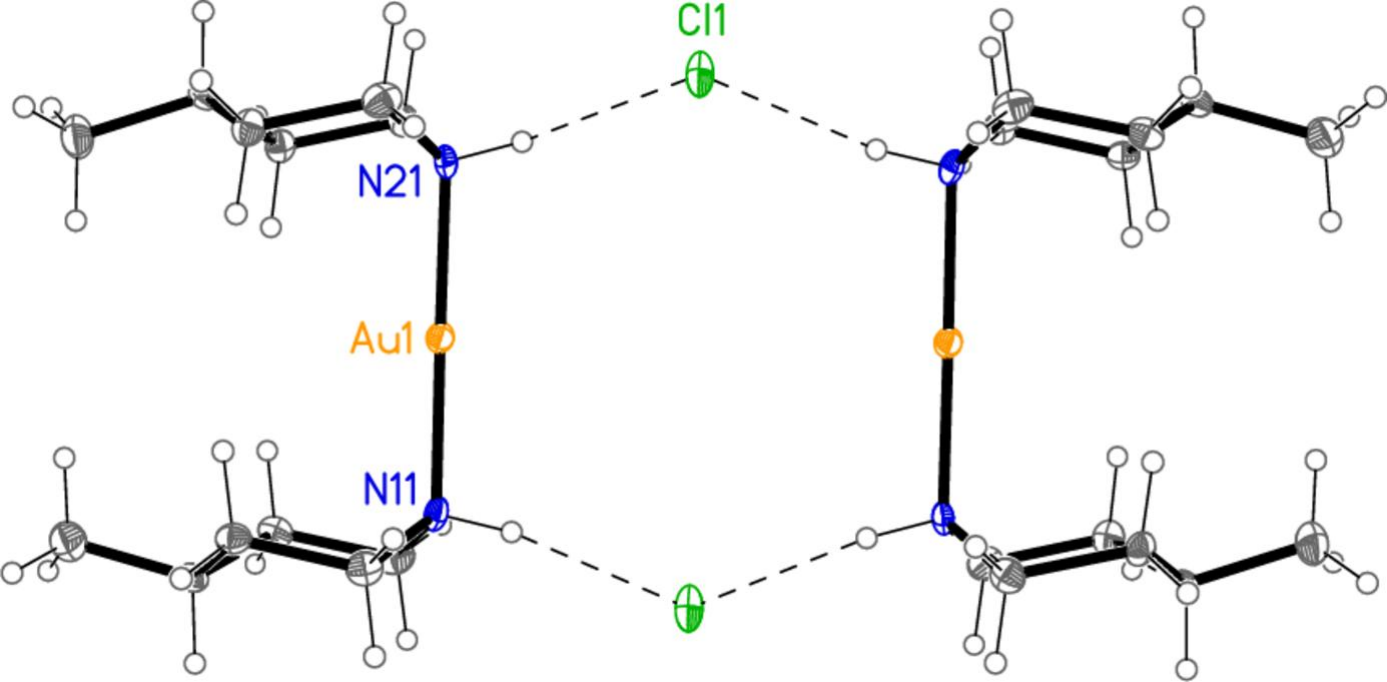
The hydrogen-bonded dimer of compound **1**. Dashed lines indicate hydrogen bonds.

**Figure 7:**
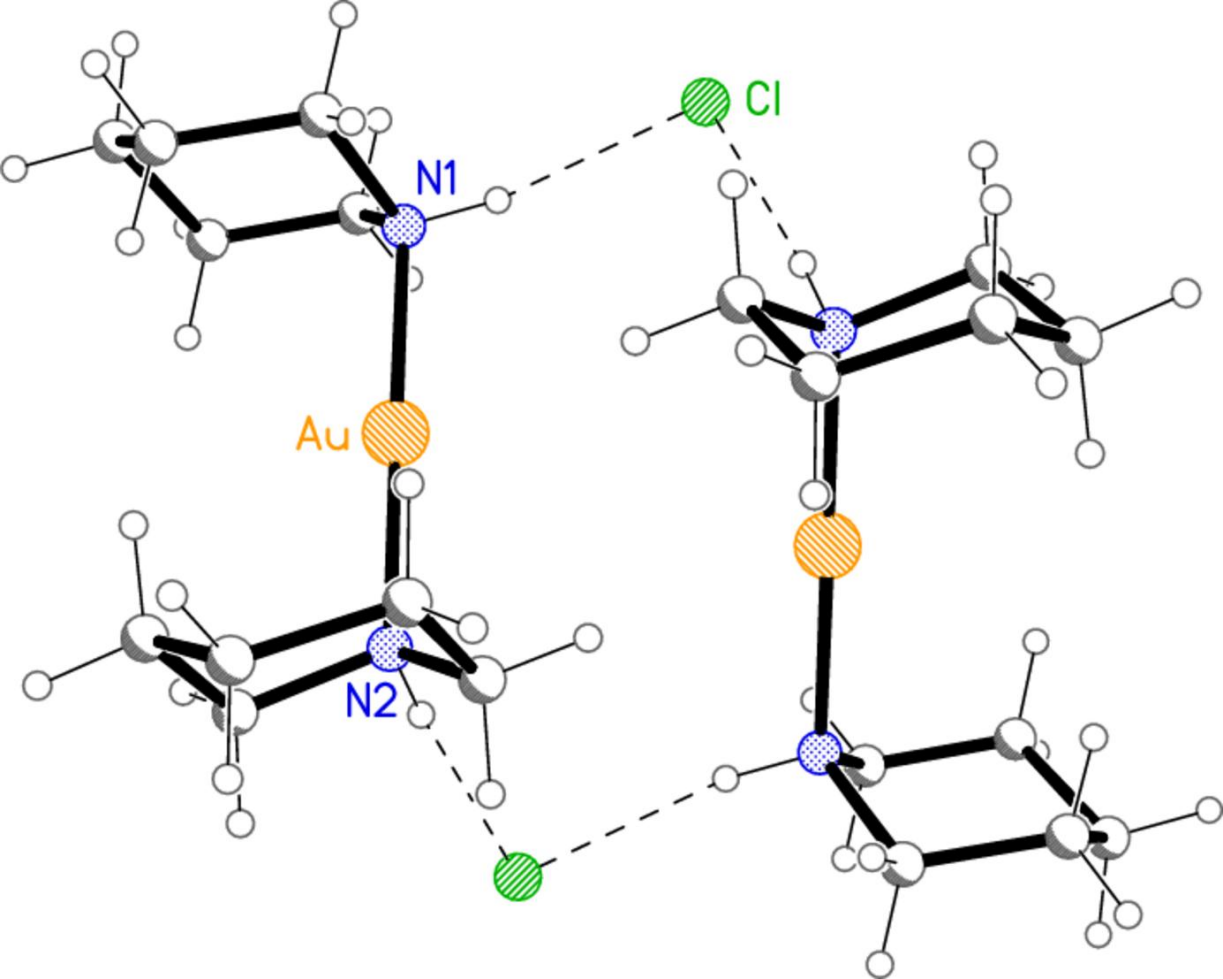
The hydrogen-bonded dimer of [Au(pip)_2_]Cl (Ahrens *et al.*, 1999 ▸), drawn from deposited coordinates. Radii are arbitrary. Dashed lines indicate hydrogen bonds.

**Figure 8:**
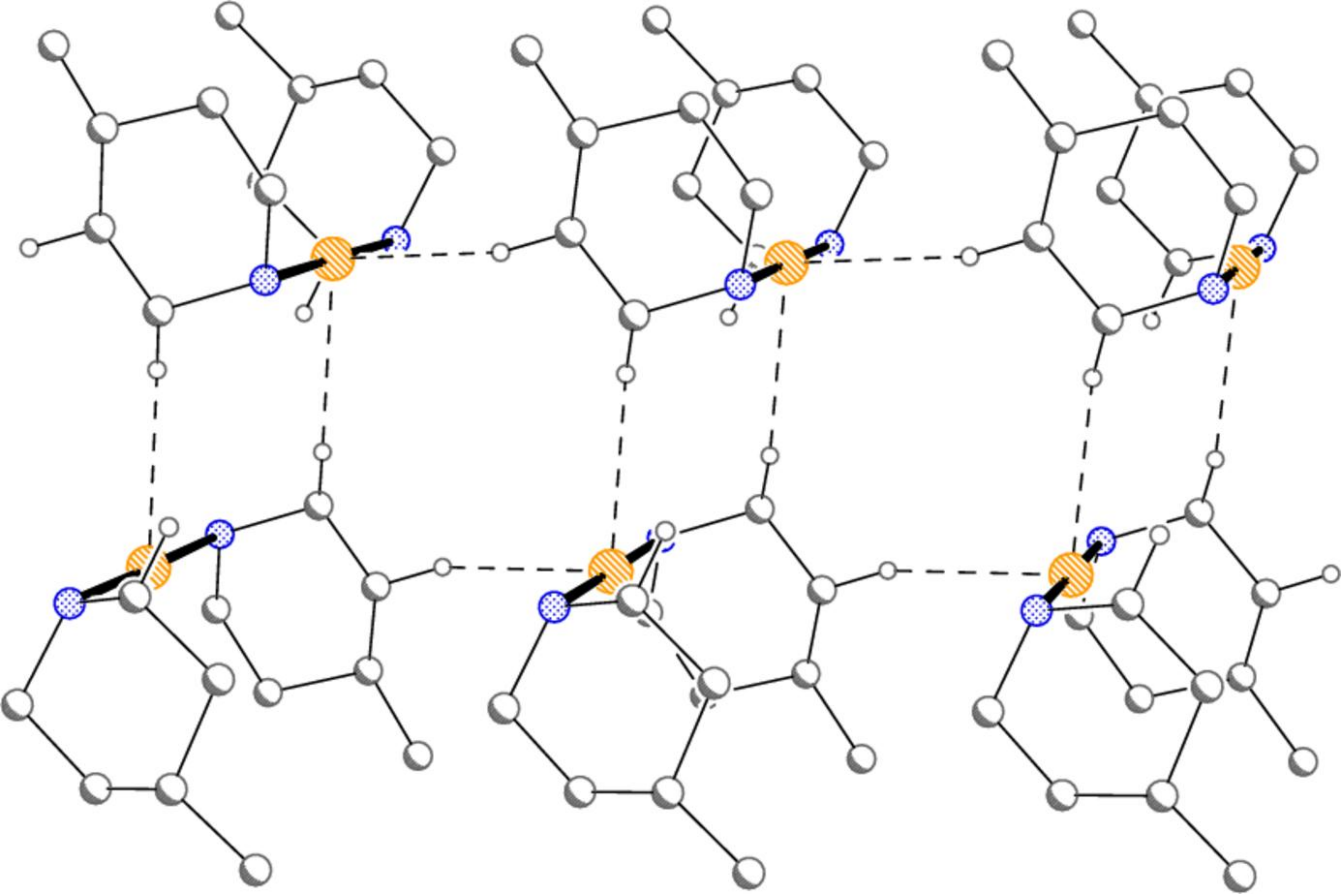
Compound **1**: The short H⋯Au contacts (dashed lines) combine to form a ribbon of cations parallel to the *a* axis. Hydrogen atoms not involved in these inter­actions are omitted.

Compound **1** forms inversion-symmetric dimers with hydrogen bonding of the form N—H⋯Cl^−^⋯H—N (Fig. 6 ▸, Table 6 ▸); the central hydrogen-bonded ring has graph set 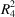 (12) (Bernstein *et al.*, 1995 ▸). The same applies to the [Au(pip)_2_]Cl dimer (Ahrens *et al.*, 1999 ▸), and yet, despite the topological similarity, there are major differences between these dimeric substructures. For **1**, the H⋯Cl^−^⋯H angle is wider at 135 (1)°, the Au⋯Au distance is much longer at 5.9269 (3) Å, and the piperidine rings are approximately eclipsed, whereas in [Au(pip)_2_]Cl (Fig. 7 ▸) the H⋯Cl^−^⋯H angle of 82° is much narrower, the Au⋯Au distance of 4.085 Å is shorter and the piperidine rings are significantly rotated around the N⋯N vector, with three absolute C—N⋯N—C pseudo torsion angles of approximately 60° and one approximately anti­periplanar (values calculated from deposited coordinates). It is tempting to suggest that the axial configuration of the Au atoms may facilitate the formation of the dimers, but detailed theoretical calculations would be necessary to provide corroborative evidence for this. Apart from the classical hydrogen bonds, three short contacts (Table 6 ▸) might be regarded as ‘weak’ hydrogen bonds. Two of these involve the gold atom as hydrogen-bond acceptor, a topic that has been reviewed by Schmidbaur *et al.* (2014 ▸) and Schmidbaur (2019 ▸), and lead to ribbons of cations parallel to the *a* axis (Fig. 8 ▸).

**Figure 9:**
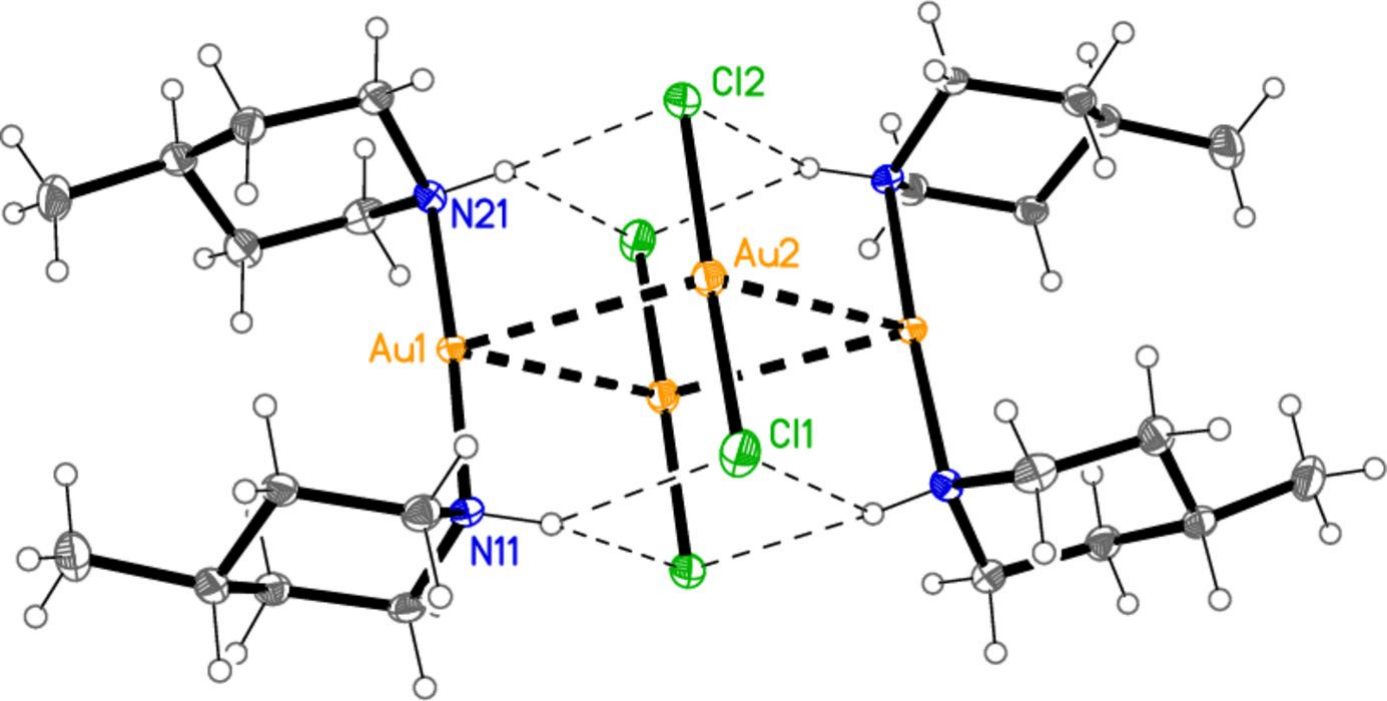
The hydrogen-bonded dimer of compound **2**. Dashed lines indicate hydrogen bonds (thin) or aurophilic inter­actions (thick). Atom labels indicate the asymmetric unit.

**Figure 10:**
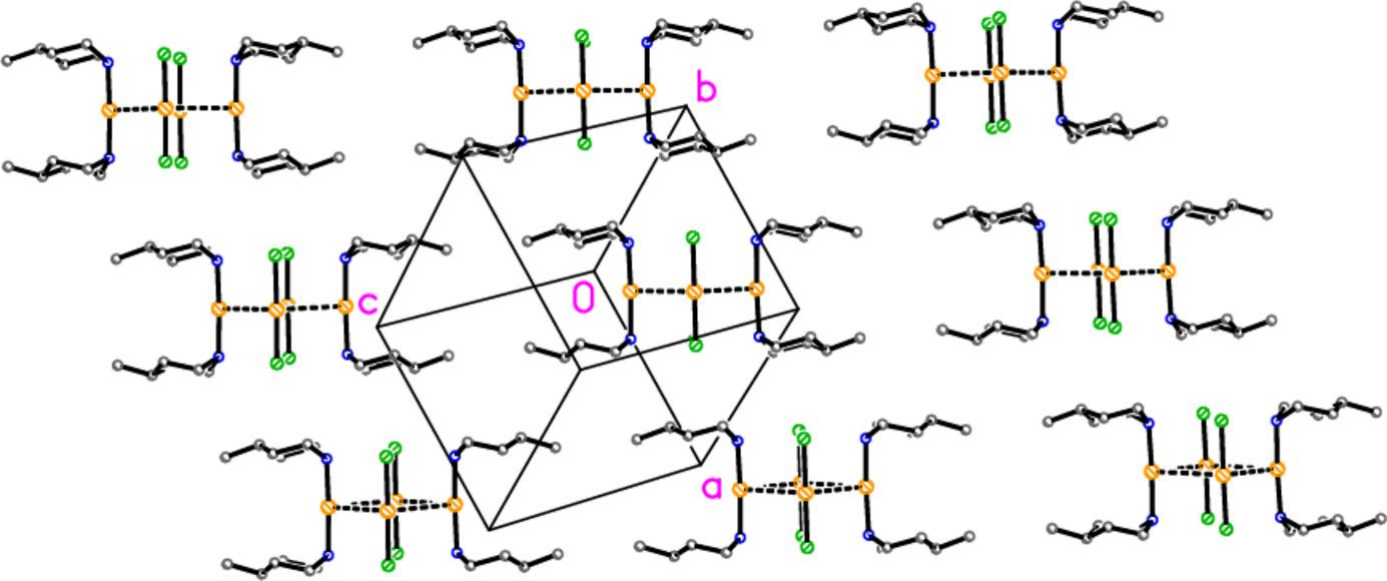
General packing of compound **2**, showing a layer parallel to (011), with view direction perpendicular to the layer. This layer passes through the region at *y* ≃ 0.25, *z* ≃ 0.25; a further such layer passes through the region at *y* ≃ 0.75, *z* ≃ 0.75.

Compound **2** also forms inversion-symmetric dimers (Fig. 9 ▸) with a quadrilateral of Au atoms, exactly planar by symmetry, connected *via* two independent aurophilic inter­actions, and two H_2_Cl_2_ quadrilaterals, above and below this plane, involving three-centre NH(⋯Cl)_2_ inter­actions (Table 7 ▸). This motif is topologically analogous to that of the AuCl(piperidine) tetra­mer (Guy *et al.*, 1977 ▸; for an improved Figure of this structure, see Döring & Jones, 2023*a* ▸). The Au_4_ quadrilateral displays widely differing angles (Table 2 ▸), whereby the transannular Au2⋯Au2′ distance is much smaller than Au1⋯Au1′ [3.5051 (8) and 5.3963 (6) Å, respectively]. The angles in the H_2_Cl_2_ quadrilateral are approximately equal [89 (3)° at the chlorine atoms and 91 (2)° at the hydrogen atoms]. There are no C—H⋯Cl or C—H⋯Au contacts shorter than 2.9 or 3.1 Å, respectively, so that one may loosely speak of a packing of dimers that involves only van der Waals inter­actions; the dimers lie in layers parallel to (011), whereby neighbouring dimers are related by translational symmetry parallel to [100] and [11 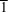 ] (approximately vertical and horizontal, respectively, in Fig. 10 ▸).

**Figure 11:**
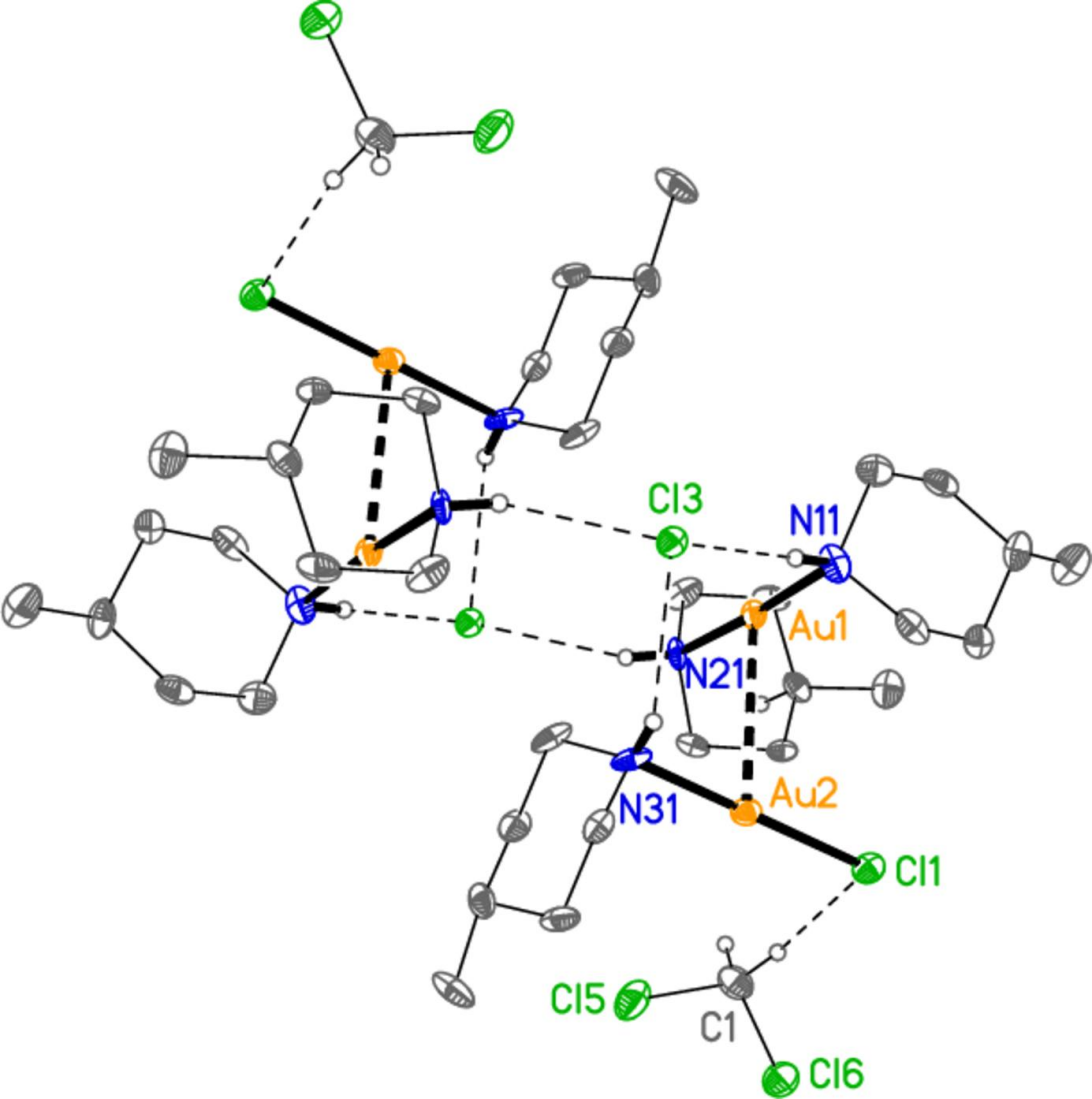
The inversion-symmetric dimer of the first formula unit of compound **4**. Dashed lines indicate hydrogen bonds (thin) or aurophilic inter­actions (thick). Atom labels indicate the asymmetric unit.

**Figure 12:**
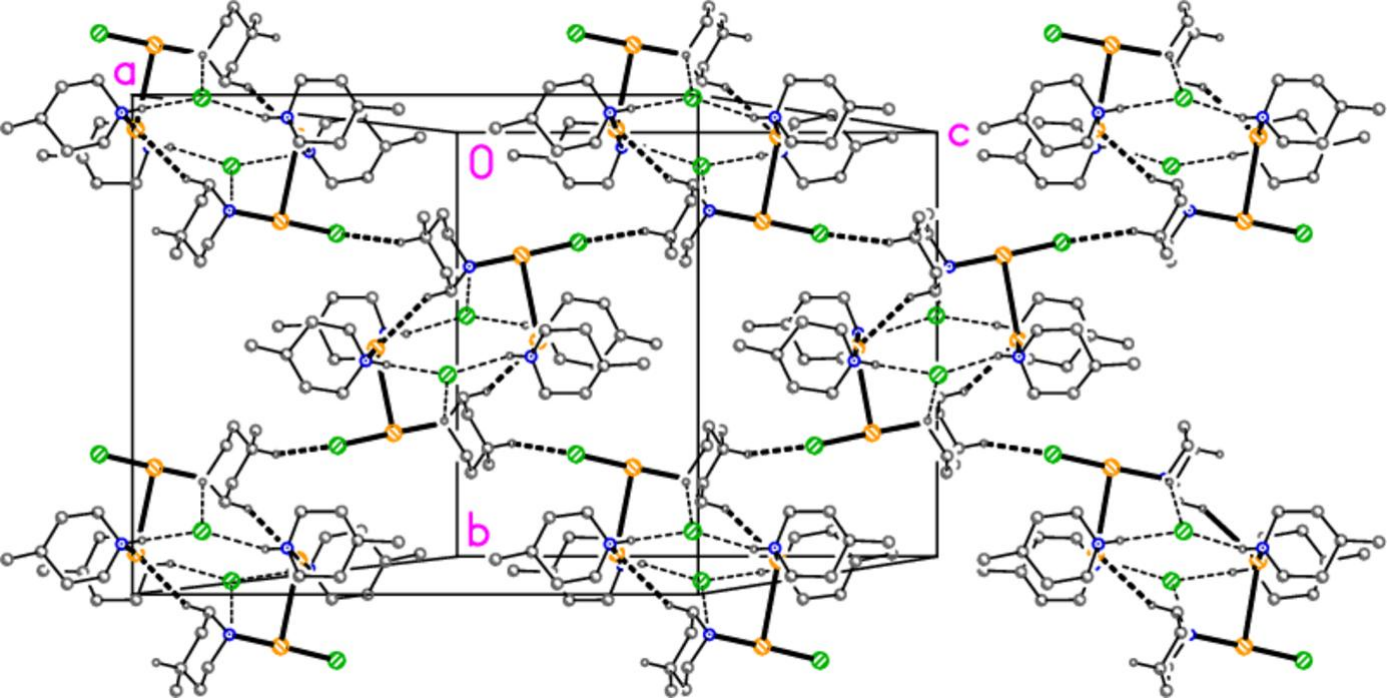
Compound **4**: Connection of the dimers of the first formula unit by the ‘weak’ hydrogen bonds of the form H⋯Au and H⋯Cl (thick dashed lines). Classical hydrogen bonds are represented by thin dashed lines. The view direction is perpendicular to the *bc* plane, and the region is *x* ≃ 0.

**Figure 13:**
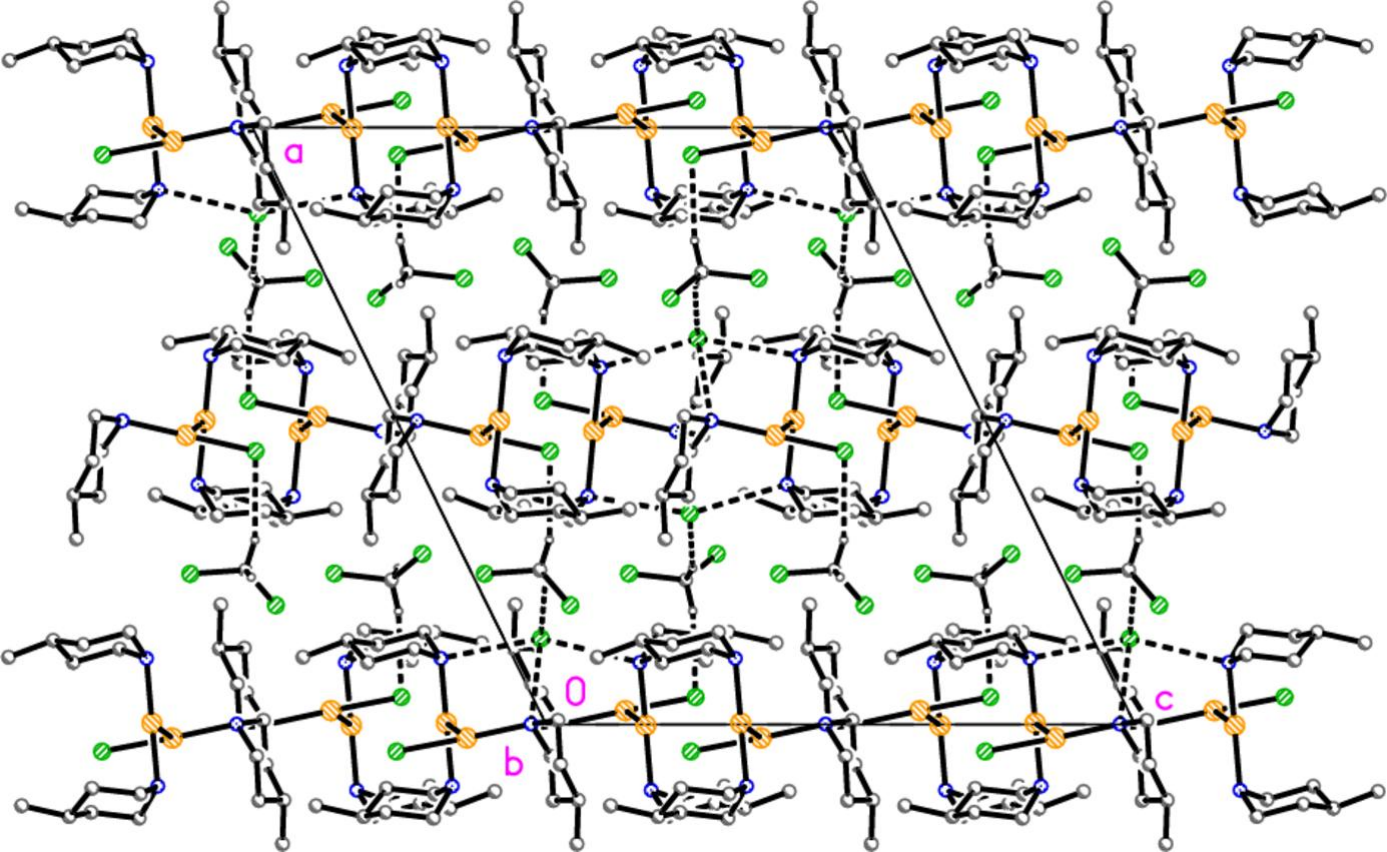
Compound **4**: Projection of the structure parallel to the *b* axis. Hydrogen atoms (except for those of the solvent) are omitted. Dashed lines indicate hydrogen bonds. The layers of gold-containing residues at *x* ≃ 0, 0.5 and 1 can be clearly recognized.

Both formula units of compound **4** form closely similar inversion-symmetric dimers *via* additional hydrogen bonds from H21 or H51 to the chloride ion (Table 9 ▸, Fig. 11 ▸). The centre of the dimer is a hydrogen-bonded ring of graph set *R* ^2^_4_(12). There are many short contacts of the type H⋯Cl or H⋯Au that might be regarded as ‘weak’ hydrogen bonds (Table 9 ▸). The contacts H34⋯Cl1 and H36*B*⋯Au1 connect the dimers of the first formula unit to form a layer parallel to the *bc* plane (Fig. 12 ▸), whereas H64⋯Cl2 and H66*A*⋯Au2 do the same for the second unit, although it is often a moot point whether short contacts to Au(I) centres are of structural significance, or whether they are simply a consequence of the sterically exposed nature of a linearly coordinated atom. A projection of the structure parallel to the *b* axis (Fig. 13 ▸) shows that the gold complexes of the first formula unit occupy the regions *x* ≃ 0 and 1, whereas those of formula unit 2 lie in the region *x* ≃ 0.5, with the solvent mol­ecules between these broad layers.

**Figure 14:**
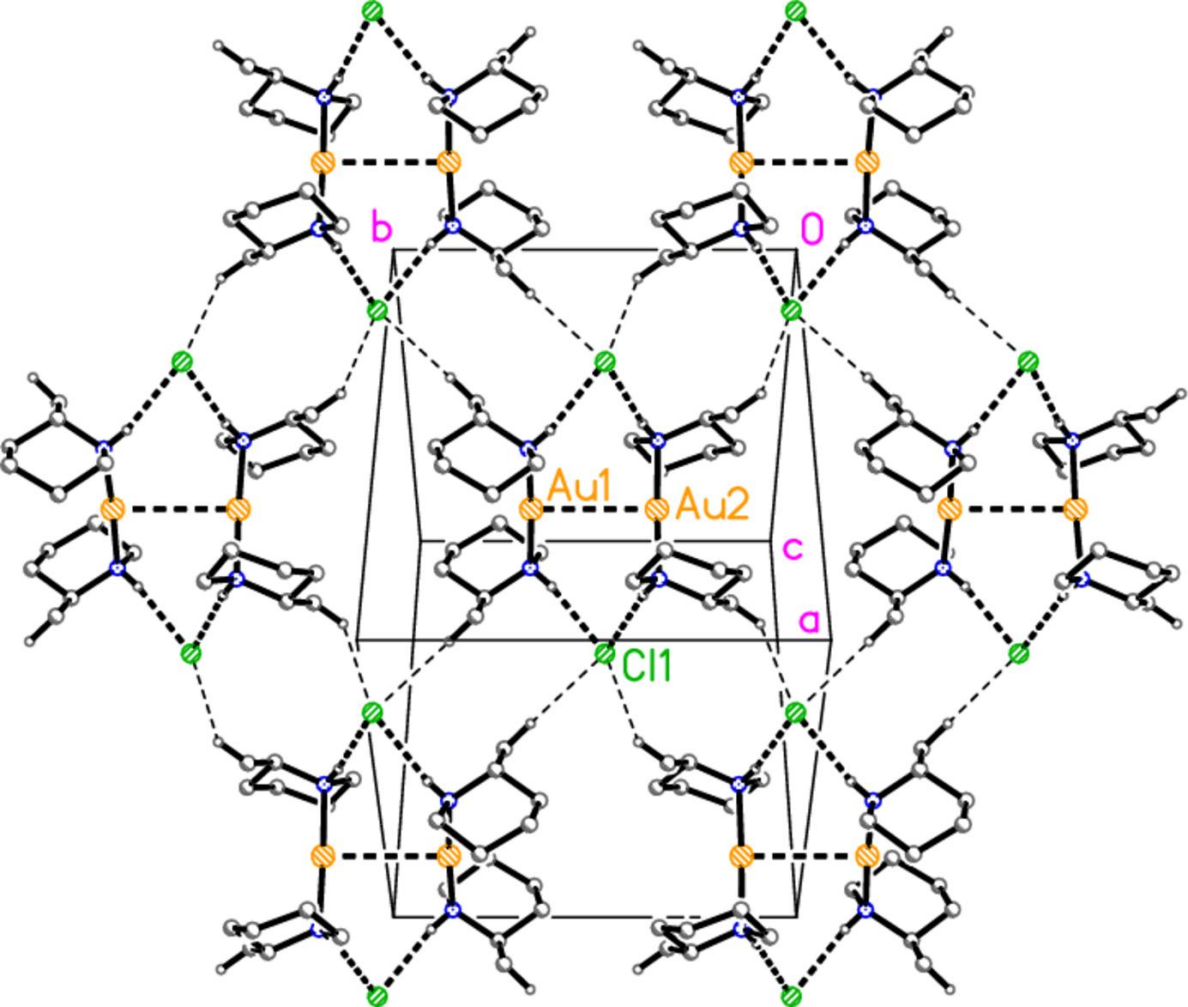
Compound **5**: Packing of the dimers *via* short H_meth­yl_⋯Cl contacts to form a hexa­gonal layer. The view direction is perpendicular to (10 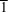 ). Dashed lines indicate (thick) classical hydrogen bonds and aurophilic inter­actions or (thin) ‘weak’ hydrogen bonds C—H⋯Cl. The centre of the layer is at (1/2, 1/2, 1/4).

The packing of the dimers of compound **5** is essentially featureless. Three of the four shortest C—H⋯Cl contacts involve methyl hydrogens (whose position is always somewhat unreliable for heavy-atom structures) and all short C—H⋯Au contacts are intra­molecular. However, the two shortest C—H⋯Cl contacts (Table 10 ▸) serve to link the dimers, forming a layer structure parallel to (10 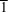 ) (Fig. 14 ▸). The hexa­gonal packing of the dimers may nevertheless be determined more by steric or van der Waals effects.

## 4. Database survey

The searches employed the routine ConQuest (Bruno *et al.*, 2002 ▸), part of Version 2022.3.0 of the CSD (Groom *et al.*, 2016 ▸).

Further examples of complexes with the type III stoichiometry, but involving imino ligands, were observed for the structures [Au(Ph_2_C=NH)_2_] [Au*X*_2_] (*X* = Cl or Br; REXRER and REXRIV; Schneider *et al.*, 1997*a* ▸) and [Au(Me_2_NC=NH)_2_] [AuBr_2_] (RIXYAY; Schneider *et al.*, 1997*b* ▸).

Few structures of transition-metal complexes involving alkyl­piperidine ligands have been reported, and most of these involved methyl substituents. For 3-Me-pip (which we did not study) there is only [Pt(malonate)(3-Me-pip)_2_], with inversion symmetry, in which both the metal and the methyl group are equatorial (QUBFOI; Khan *et al.*, 2000 ▸). For 2-Me-pip there are two structures: in the structure of enanti­omerically pure [W(CO)_5_(2-Me-pip)], with *S*,*S* configuration at C2 and the nitro­gen atom (CAPSOB; Korp *et al.*, 1983 ▸), the metal and the methyl group are also equatorial, and this is also the case for the cubane-type tetra­mer [CuI(2-Me-pip)]_4_ (ZAYYAD; Wang *et al.*, 2022 ▸).

Nine complexes of 4-Me-pip appear in the CSD, seven of which display the usual equatorial positions of the metal atoms and methyl groups. The exceptions, with equatorial methyl groups but axially positioned metal atoms, are provided by two silver complexes studied by us (Jones & Wölper, 1975 ▸; Wölper *et al.*, 2010 ▸), namely [(AgCl)_5_(4-Me-pip)_4_] (GAQLEQ) and the polymeric [(AgBr)_3_(4-Me-pip)_2_] (YUXWOE), which contain one and six independent ligands, respectively. This reinforces our observation that coinage metals have a higher tendency to be axial at piperidine ligands. In this context, the 4-benzyl­piperidine complex [(AgCN)_2_(4-Bz-pip)] (CITWOU; Bz = benzyl; Strey & Döring, 2018 ▸) is inter­esting; the silver atom is axial at one of the two independent ligands but equatorial at the other.

## 5. Synthesis and crystallization

*Bis(4-methyl­piperidine)­gold(I) chloride* **1**. 40 mg (0.125 mmol) of AuCl(tht) (tht = tetra­hydro­thio­phene) were dissolved in 2 mL of 4-methyl­piperidine. The solution was divided into five portions in small test-tubes and overlaid with various precipitants [see Döring and Jones (2023*a* ▸) for details], before being stoppered and stored in a refrigerator overnight. The portion with petroleum ether as precipitant yielded crystals in the form of colourless blocks, one of which was used for the structure analysis, in approximately qu­anti­tative yield. Analysis: calculated: C 33.46, H 6.08, N 6.50; found: C 33.09, H 5.94, N 6.33%.

*Bis(4-methyl­piperidine)­gold(I) di­chlorido­aurate(I)* **2**. 40 mg (0.093 mmol) of **1** were dissolved in 2 mL of di­chloro­methane. The solution was then treated as above for **1**. The portion with *n*-pentane as precipitant yielded crystals in the form of colourless blocks, one of which was used for the structure analysis, in approximately 90% yield. Analysis: calculated: C 21.73, H 3.95, N 4.22; found: C 22.07, H 4.01, N 4.11%.

*Bis(4-methyl­piperidine)­gold(I) di­bromido­aurate(I)* **3**. 90 mg (0.247 mmol) of AuBr(tht) were dissolved in 2 mL of 4-methyl­piperidine. The solution was then treated as above for **1**. The portion with *n*-pentane as precipitant yielded crystals in the form of colourless blocks, one of which was used for the structure analysis, in approximately qu­anti­tative yield. Analysis: calculated: C 19.16, H 3.48, N 3.73; found: C 19.38, H 3.55, N 3.57%.

*Chlorido­(4-methyl­piperidine)­gold(I) bis­(4-methyl­pip­eri­dine)­gold(I) chloride di­chloro­methane solvate* **4**. 124.4 mg (0.288 mmol) of **1** were dissolved in 2 mL of di­chloro­methane and overlayered with *n*-pentane in a 100 mL round-bottomed flask, which was stoppered and stored in a refrigerator, whereby colourless triangular plates were obtained (yield not measured).

*Bis(2-methyl­piperidine)­gold(I) chloride* **5**. 40 mg (0.125 mmol) of AuCl(tht) were dissolved in 2 mL of 2-methyl­piperidine. The solution was then treated as above for **1**. The portion with *n*-pentane as precipitant yielded crystals in the form of colourless blocks, one of which was used for the structure analysis, in approximately qu­anti­tative yield. Analysis: calculated: C 33.46, H 6.08, N 6.50; found: C 32.99, H 6.26, N 6.22%.

## 6. Refinement

Details of the measurements and refinements are given in Table 11 ▸. Structures were refined anisotropically on *F* ^2^. Methyl­ene hydrogens were included at calculated positions and refined using a riding model with C—H = 0.99 Å and H—C—H = 109.5°. Methine hydrogens were included similarly, but with C—H = 0.99 Å. Methyl groups were included as idealized rigid groups with C—H = 0.98 Å and H—C—H = 109.5°, and were allowed to rotate but not tip. *U* values of the hydrogen atoms were fixed at 1.5 × *U*_eq_ of the parent carbon atoms for methyl groups and 1.2 × *U*_eq_ of the parent carbon atoms for other hydrogens.

For all compounds, the NH hydrogen atoms were refined freely but with N—H distances restrained to be approximately equal. For compound **4**, *U* values of the NH hydrogen atoms were fixed at 1.2 × *U*_eq_ of the parent nitro­gen atoms, because the values were otherwise too small (close to or slightly below zero).

The crystal of compound **2** was pseudo-merohedrally twinned by inter­change of the *a* and *b* axes, with the twin matrix [0 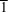 0 / 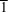 00 / 00 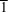 ]. The relative volume of the smaller component refined to 0.3023 (7). Five badly-fitting reflections were omitted from the refinement.

For compound **3**, the cell is presented in a non-standard form (*b* > *a*) in order to allow a direct comparison with the isotypic chlorine analogue **2**.

The crystal of compound **4** was pseudo-merohedrally twinned (*via* an apparently ortho­rhom­bic cell) with the twin matrix [ 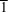 0 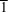 / 0 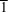 0 / 001]. The relative volume of the smaller component refined to 0.4614 (5). Six badly-fitting reflections were omitted from the refinement.

For compound **5**, the *U* values are rather high for a structure measured at 100 K. Accordingly, Fig. 5 ▸ shows ellipsoids at the 30% level.

## Supplementary Material

Crystal structure: contains datablock(s) 1, 2, 3, 4, 5, global. DOI: 10.1107/S2056989023010940/yz2046sup1.cif

Structure factors: contains datablock(s) 1. DOI: 10.1107/S2056989023010940/yz20461sup2.hkl

Structure factors: contains datablock(s) 2. DOI: 10.1107/S2056989023010940/yz20462sup3.hkl

Structure factors: contains datablock(s) 3. DOI: 10.1107/S2056989023010940/yz20463sup4.hkl

Structure factors: contains datablock(s) 4. DOI: 10.1107/S2056989023010940/yz20464sup5.hkl

Structure factors: contains datablock(s) 5. DOI: 10.1107/S2056989023010940/yz20465sup6.hkl

CCDC references: 2113942, 2113943, 2113944, 2113945, 2113941

Additional supporting information:  crystallographic information; 3D view; checkCIF report

## Figures and Tables

**Table 1:** Selected geometric parameters (Å, °) for **1** [Chem scheme1]

Au1—N11	2.051 (3)	Au1—N21	2.052 (3)
			
N11—Au1—N21	179.43 (12)		
			
Au1—N11—C12—C13	−67.1 (3)	Au1—N21—C22—C23	−65.0 (3)
C12—C13—C14—C17	175.6 (3)	C22—C23—C24—C27	173.6 (3)
C17—C14—C15—C16	−176.5 (3)	C27—C24—C25—C26	−173.4 (3)
Au1—N11—C16—C15	67.2 (3)	Au1—N21—C26—C25	64.9 (3)

**Table 2:** Selected geometric parameters (Å, °) for **2** [Chem scheme1]

Au1—N21	2.062 (7)	Au1—Au2^i^	3.2252 (4)
Au1—N11	2.064 (6)	Au2—Cl1	2.282 (2)
Au1—Au2	3.2096 (5)	Au2—Cl2	2.282 (2)
			
N21—Au1—N11	176.8 (2)	Cl1—Au2—Cl2	173.42 (7)
N21—Au1—Au2	95.61 (17)	Cl1—Au2—Au1	96.01 (5)
N11—Au1—Au2	87.59 (17)	Cl2—Au2—Au1	87.38 (5)
N21—Au1—Au2^i^	87.50 (18)	Cl1—Au2—Au1^i^	95.47 (5)
N11—Au1—Au2^i^	94.13 (17)	Cl2—Au2—Au1^i^	88.25 (5)
Au2—Au1—Au2^i^	66.011 (12)	Au1—Au2—Au1^i^	113.989 (13)
			
Au1—N11—C12—C13	−66.9 (7)	Au1—N21—C22—C23	66.3 (7)
C12—C13—C14—C17	175.7 (7)	C22—C23—C24—C27	−174.6 (7)
C17—C14—C15—C16	−175.9 (7)	C27—C24—C25—C26	174.6 (7)
Au1—N11—C16—C15	66.4 (7)	Au1—N21—C26—C25	−65.7 (7)

Symmetry code: (i) 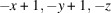 .

**Table 3:** Selected geometric parameters (Å, °) for **3** [Chem scheme1]

Au1—N11	2.062 (3)	Au1—Au2^i^	3.3094 (2)
Au1—N21	2.064 (3)	Au2—Br2	2.4006 (4)
Au1—Au2	3.2988 (3)	Au2—Br1	2.4027 (4)
			
N11—Au1—N21	176.30 (13)	Br2—Au2—Br1	170.628 (15)
			
Au1—N11—C12—C13	−67.5 (3)	Au1—N21—C22—C23	67.3 (3)
C12—C13—C14—C17	175.4 (3)	C22—C23—C24—C27	−174.6 (3)
C17—C14—C15—C16	−176.1 (3)	C27—C24—C25—C26	174.0 (3)
Au1—N11—C16—C15	67.4 (3)	Au1—N21—C26—C25	−66.5 (3)

Symmetry code: (i) 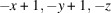 .

**Table 4:** Selected geometric parameters (Å, °) for **4** [Chem scheme1]

Au1—N21	2.042 (8)	Au3—N41	2.051 (8)
Au1—N11	2.061 (8)	Au3—N51	2.060 (9)
Au1—Au2	3.3138 (6)	Au3—Au4	3.2619 (5)
Au2—N31	2.078 (9)	Au4—N61	2.062 (9)
Au2—Cl1	2.267 (3)	Au4—Cl2	2.261 (3)
			
N21—Au1—N11	176.2 (4)	N41—Au3—N51	175.4 (3)
N21—Au1—Au2	97.4 (2)	N41—Au3—Au4	88.2 (3)
N11—Au1—Au2	86.2 (3)	N51—Au3—Au4	96.1 (2)
N31—Au2—Cl1	178.1 (2)	N61—Au4—Cl2	178.1 (3)
N31—Au2—Au1	88.0 (2)	N61—Au4—Au3	87.3 (2)
Cl1—Au2—Au1	93.18 (6)	Cl2—Au4—Au3	94.52 (6)
			
Au1—N11—C12—C13	−67.1 (10)	Au3—N41—C42—C43	67.3 (10)
C12—C13—C14—C17	174.6 (9)	C42—C43—C44—C47	−174.5 (9)
C17—C14—C15—C16	−173.3 (8)	C47—C44—C45—C46	174.3 (8)
Au1—N11—C16—C15	63.9 (9)	Au3—N41—C46—C45	−64.0 (10)
Au1—N21—C22—C23	67.9 (9)	Au3—N51—C52—C53	−68.4 (10)
C22—C23—C24—C27	−173.0 (9)	C52—C53—C54—C57	173.2 (9)
C27—C24—C25—C26	174.0 (8)	C57—C54—C55—C56	−174.7 (9)
Au1—N21—C26—C25	−66.4 (9)	Au3—N51—C56—C55	66.3 (10)
Au2—N31—C32—C33	−66.5 (9)	Au4—N61—C62—C63	65.8 (10)
C32—C33—C34—C37	175.4 (9)	C62—C63—C64—C67	−173.6 (10)
C37—C34—C35—C36	−175.3 (9)	C67—C64—C65—C66	173.0 (9)
Au2—N31—C36—C35	65.4 (9)		

**Table 5:** Selected geometric parameters (Å, °) for **5** [Chem scheme1]

Au1—N11	2.053 (3)	Au2—N21	2.057 (3)
Au1—Au2	3.3854 (3)		
			
N11^i^—Au1—N11	176.9 (2)	N21—Au2—Au1	91.11 (11)
N11—Au1—Au2	91.54 (10)	N21^i^—Au2—Au1	91.11 (11)
			
C16—N11—C12—C17	176.6 (4)	C26—N21—C22—C27	174.5 (4)
Au1—N11—C12—C13	70.4 (4)	Au2—N21—C22—C23	69.6 (4)
Au1—N11—C16—C15	−67.9 (4)	Au2—N21—C26—C25	−67.5 (4)

Symmetry code: (i) 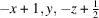 .

**Table 6:** Hydrogen-bond geometry (Å, °) for **1** [Chem scheme1]

*D*—H⋯*A*	*D*—H	H⋯*A*	*D*⋯*A*	*D*—H⋯*A*
N21—H02⋯Cl1	0.92 (3)	2.23 (3)	3.144 (3)	171 (3)
N11—H01⋯Cl1^i^	0.90 (3)	2.25 (3)	3.140 (3)	170 (4)
C16—H16*A*⋯Cl1^ii^	0.99	2.85	3.623 (4)	136
C22—H22*B*⋯Au1^ii^	0.99	2.80	3.724 (4)	155
C23—H23*A*⋯Au1^iii^	0.99	2.85	3.685 (4)	143

Symmetry codes: (i) 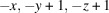 ; (ii) 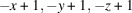 ; (iii) 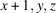 .

**Table 7:** Hydrogen-bond geometry (Å, °) for **2** [Chem scheme1]

*D*—H⋯*A*	*D*—H	H⋯*A*	*D*⋯*A*	*D*—H⋯*A*
N11—H01⋯Cl1	0.95 (6)	2.59 (7)	3.370 (6)	139 (7)
N11—H01⋯Cl2^i^	0.95 (6)	2.66 (8)	3.312 (7)	126 (6)
N21—H02⋯Cl2	0.95 (6)	2.61 (9)	3.316 (7)	131 (8)
N21—H02⋯Cl1^i^	0.95 (6)	2.66 (9)	3.360 (7)	130 (8)

Symmetry code: (i) 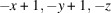 .

**Table 8:** Hydrogen-bond geometry (Å, °) for **3** [Chem scheme1]

*D*—H⋯*A*	*D*—H	H⋯*A*	*D*⋯*A*	*D*—H⋯*A*
N11—H01⋯Br2^i^	0.90 (3)	2.80 (4)	3.457 (3)	131 (4)
N11—H01⋯Br1	0.90 (3)	2.81 (4)	3.518 (3)	136 (4)
N21—H02⋯Br1^i^	0.89 (3)	2.77 (4)	3.487 (3)	138 (4)
N21—H02⋯Br2	0.89 (3)	2.84 (4)	3.462 (3)	128 (4)

Symmetry code: (i) 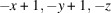 .

**Table 9:** Hydrogen-bond geometry (Å, °) for **4** [Chem scheme1]

*D*—H⋯*A*	*D*—H	H⋯*A*	*D*⋯*A*	*D*—H⋯*A*
N11—H11⋯Cl3	0.82 (3)	2.38 (4)	3.195 (9)	178 (11)
N21—H21⋯Cl3^i^	0.81 (3)	2.45 (5)	3.229 (8)	161 (9)
N31—H31⋯Cl3	0.82 (3)	2.55 (6)	3.238 (8)	143 (8)
N41—H41⋯Cl4	0.81 (3)	2.43 (4)	3.234 (8)	172 (10)
N51—H51⋯Cl4^ii^	0.81 (3)	2.42 (4)	3.209 (9)	165 (10)
N61—H61⋯Cl4	0.81 (3)	2.50 (6)	3.237 (9)	151 (9)
C1—H1*B*⋯Cl1	0.99	2.66	3.638 (11)	169
C1—H1*A*⋯Cl4	0.99	2.51	3.432 (11)	155
C2—H2*A*⋯Cl2	0.99	2.75	3.710 (11)	162
C2—H2*B*⋯Cl3^iii^	0.99	2.59	3.431 (10)	143
C36—H36*B*⋯Au1^i^	0.99	2.87	3.770 (9)	152
C16—H16*A*⋯Au2	0.99	2.71	3.562 (10)	144
C66—H66*A*⋯Au3^ii^	0.99	2.91	3.778 (9)	147
C46—H46*B*⋯Au4	0.99	2.75	3.579 (11)	142
C34—H34⋯Cl1^iv^	1.00	2.86	3.801 (13)	156
C64—H64⋯Cl2^v^	1.00	2.81	3.755 (14)	158

Symmetry codes: (i) 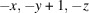 ; (ii) 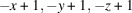 ; (iii) 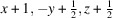 ; (iv) 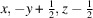 ; (v) 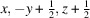 .

**Table 10:** Hydrogen-bond geometry (Å, °) for **5** [Chem scheme1]

*D*—H⋯*A*	*D*—H	H⋯*A*	*D*⋯*A*	*D*—H⋯*A*
N11—H01⋯Cl1	0.80 (3)	2.34 (3)	3.110 (4)	162 (4)
N21—H02⋯Cl1	0.80 (3)	2.36 (3)	3.149 (4)	170 (5)

**Table 11:** Experimental details

	**1**	**2**	**3**	**4**	**5**
Crystal data					
Chemical formula	[Au(C_6_H_13_N)_2_]Cl	[Au(C_6_H_13_N)_2_][AuCl_2_]	[Au(C_6_H_13_N)_2_][AuBr_2_]	[Au(C_6_H_13_N)_2_]Cl·[AuCl(C_6_H_13_N)]·CH_2_Cl_2_	[Au(C_6_H_13_N)_2_]Cl
*M*_r_	430.76	663.18	752.10	847.28	430.76
Crystal system, space group	Monoclinic, *P*2_1_/*n*	Triclinic, *P* 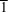	Triclinic, *P* 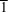	Monoclinic, *P*2_1_/*c*	Monoclinic, *C*2/*c*
Temperature (K)	100	100	100	101	100
*a*, *b*, *c* (Å)	6.4068 (3), 25.2542 (15), 9.3395 (4)	9.6998 (7), 9.7001 (8), 10.7194 (5)	9.8461 (6), 9.7728 (4), 10.9461 (5)	20.5785 (7), 16.0876 (4), 18.2247 (7)	17.6978 (7), 11.2748 (5), 16.5620 (6)
α, β, γ (°)	90, 103.946 (4), 90	102.218 (6), 101.893 (5), 114.695 (8)	100.136 (4), 103.685 (5), 116.287 (5)	90, 116.196 (5), 90	90, 114.013 (5), 90
*V* (Å^3^)	1466.57 (12)	844.89 (11)	868.59 (9)	5413.7 (4)	3018.8 (2)
*Z*	4	2	2	8	8
Radiation type	Mo *K*α	Mo *K*α	Mo *K*α	Mo *K*α	Mo *K*α
μ (mm^−1^)	10.19	17.65	21.46	11.23	9.90
Crystal size (mm)	0.21 × 0.03 × 0.01	0.15 × 0.03 × 0.03	0.08 × 0.06 × 0.05	0.25 × 0.1 × 0.1	0.2 × 0.2 × 0.1

Data collection					
Diffractometer	Oxford Diffraction Xcalibur, Eos	Oxford Diffraction Xcalibur, Eos	Oxford Diffraction Xcalibur, Eos	Oxford Diffraction Xcalibur, Eos	Oxford Diffraction Xcalibur, Eos
Absorption correction	Multi-scan (*CrysAlis PRO*; Rigaku OD, 2020 ▸)	Multi-scan (*CrysAlis PRO*; Rigaku OD, 2020 ▸)	Multi-scan (*CrysAlis PRO*; Rigaku OD, 2020 ▸)	Multi-scan (*CrysAlis PRO*; Rigaku OD, 2020 ▸)	Multi-scan (*CrysAlis PRO*; Rigaku OD, 2020 ▸)
*T*_min_, *T*_max_	0.398, 1.000	0.388, 1.000	0.576, 1.000	0.580, 1.000	0.472, 1.000
No. of measured, independent and observed [*I* > 2σ(*I*)] reflections	49187, 4248, 3650	54252, 4874, 4690	48187, 5188, 4466	134776, 13423, 11445	56753, 4576, 3012
*R*_int_	0.068	0.080	0.050	0.122	0.051
(sin θ/λ)_max_ (Å^−1^)	0.704	0.704	0.724	0.667	0.724

Refinement					
*R*[*F* ^2^ > 2σ(*F* ^2^)], *wR*(*F* ^2^), *S*	0.033, 0.048, 1.16	0.029, 0.064, 1.13	0.023, 0.041, 1.05	0.040, 0.070, 1.09	0.029, 0.049, 1.09
No. of reflections	4248	4874	5188	13423	4576
No. of parameters	155	174	173	530	156
No. of restraints	1	1	1	15	1
H-atom treatment	H atoms treated by a mixture of independent and constrained refinement	H atoms treated by a mixture of independent and constrained refinement	H atoms treated by a mixture of independent and constrained refinement	H atoms treated by a mixture of independent and constrained refinement	H atoms treated by a mixture of independent and constrained refinement
Δρ_max_, Δρ_min_ (e Å^−3^)	1.13, −1.57	2.02, −2.52	1.02, −1.15	2.02, −1.92	2.19, −0.93

Computer programs: *CrysAlis PRO* (Rigaku OD, 2020 ▸), *SHELXS97* (Sheldrick, 2008 ▸), *SHELXL2018/3* and *SHELXL2019/3* (Sheldrick, 2015 ▸) and *XP* (Siemens, 1994 ▸).

## supplementary crystallographic information

### Bis(4-methylpiperidine-κN)gold(I) chloride (1) Crystal data

**Table:**

[Au(C_6_H_13_N)_2_]Cl	*F*(000) = 832
*M**_r_* = 430.76	*D*_x_ = 1.951 Mg m^−^^3^
Monoclinic, *P*2_1_/*n*	Mo *K*α radiation, λ = 0.71073 Å
*a* = 6.4068 (3) Å	Cell parameters from 8714 reflections
*b* = 25.2542 (15) Å	θ = 2.2–30.9°
*c* = 9.3395 (4) Å	µ = 10.19 mm^−^^1^
β = 103.946 (4)°	*T* = 100 K
*V* = 1466.57 (12) Å^3^	Lath, colourless
*Z* = 4	0.21 × 0.03 × 0.01 mm

### Bis(4-methylpiperidine-κN)gold(I) chloride (1) Data collection

**Table:**

Oxford Diffraction Xcalibur, Eos diffractometer	4248 independent reflections
Radiation source: Enhance (Mo) X-ray Source	3650 reflections with *I* > 2σ(*I*)
Graphite monochromator	*R*_int_ = 0.068
Detector resolution: 16.1419 pixels mm^-1^	θ_max_ = 30.0°, θ_min_ = 2.4°
ω scan	*h* = −9→8
Absorption correction: multi-scan (CrysAlisPro; Rigaku OD, 2020)	*k* = −35→35
*T*_min_ = 0.398, *T*_max_ = 1.000	*l* = −13→12
49187 measured reflections	

### Bis(4-methylpiperidine-κN)gold(I) chloride (1) Refinement

**Table:**

Refinement on *F*^2^	Primary atom site location: structure-invariant direct methods
Least-squares matrix: full	Secondary atom site location: difference Fourier map
*R*[*F*^2^ > 2σ(*F*^2^)] = 0.033	Hydrogen site location: mixed
*wR*(*F*^2^) = 0.048	H atoms treated by a mixture of independent and constrained refinement
*S* = 1.16	*w* = 1/[σ^2^(*F*_o_^2^) + (0.0141*P*)^2^ + 1.4591*P*] where *P* = (*F*_o_^2^ + 2*F*_c_^2^)/3
4248 reflections	(Δ/σ)_max_ < 0.001
155 parameters	Δρ_max_ = 1.13 e Å^−^^3^
1 restraint	Δρ_min_ = −1.57 e Å^−^^3^

### Bis(4-methylpiperidine-κN)gold(I) chloride (1) Special details

**Table:**

Geometry. Additional structural data: Distance 5.9269 (0.0003) Au1 - Au1_$1 Angle 135.26 ( 1.44) H02 - Cl1 - H01_$1 Torsion angles 2.93 ( 0.32) C26 - N21 - N11 - C12 2.19 ( 0.31) C22 - N21 - N11 - C16 Operator for generating equivalent atoms: $1 -x, -y+1, -z+1

### Bis(4-methylpiperidine-κN)gold(I) chloride (1) Fractional atomic coordinates and isotropic or equivalent isotropic displacement parameters (Å^2^)

**Table:**

	*x*	*y*	*z*	*U*_iso_*/*U*_eq_	
Au1	0.27850 (2)	0.58105 (2)	0.41086 (2)	0.01228 (4)	
Cl1	0.07333 (15)	0.42372 (4)	0.25393 (10)	0.01992 (19)	
N11	0.2215 (5)	0.63222 (12)	0.5676 (3)	0.0128 (6)	
H01	0.139 (6)	0.6124 (15)	0.613 (4)	0.018 (11)*	
C12	0.0986 (6)	0.68027 (15)	0.5052 (4)	0.0161 (8)	
H12A	0.058954	0.700496	0.585531	0.019*	
H12B	−0.035734	0.669534	0.434086	0.019*	
C13	0.2293 (6)	0.71521 (14)	0.4282 (4)	0.0142 (7)	
H13A	0.145689	0.747515	0.391848	0.017*	
H13B	0.255772	0.696126	0.341629	0.017*	
C14	0.4460 (6)	0.73133 (13)	0.5300 (4)	0.0132 (7)	
H14	0.415702	0.754449	0.609509	0.016*	
C15	0.5635 (6)	0.68186 (14)	0.6033 (4)	0.0140 (7)	
H15A	0.609429	0.660305	0.527799	0.017*	
H15B	0.694185	0.692767	0.677764	0.017*	
C16	0.4233 (6)	0.64834 (14)	0.6774 (4)	0.0147 (7)	
H16A	0.503518	0.616358	0.720715	0.018*	
H16B	0.385848	0.668775	0.758154	0.018*	
C17	0.5835 (7)	0.76300 (15)	0.4479 (4)	0.0220 (8)	
H17A	0.719934	0.772491	0.516224	0.033*	
H17B	0.506738	0.795302	0.407423	0.033*	
H17C	0.612007	0.741537	0.367261	0.033*	
N21	0.3334 (5)	0.52931 (12)	0.2545 (3)	0.0130 (6)	
H02	0.263 (6)	0.4985 (13)	0.266 (4)	0.013 (10)*	
C22	0.5644 (6)	0.51457 (15)	0.2782 (4)	0.0179 (8)	
H22A	0.580427	0.486931	0.206434	0.021*	
H22B	0.617710	0.499995	0.378789	0.021*	
C23	0.6963 (6)	0.56314 (14)	0.2590 (4)	0.0153 (7)	
H23A	0.848096	0.552454	0.270366	0.018*	
H23B	0.691474	0.589011	0.337743	0.018*	
C24	0.6152 (6)	0.58985 (13)	0.1087 (4)	0.0136 (7)	
H24	0.644492	0.565394	0.031584	0.016*	
C25	0.3730 (6)	0.59872 (14)	0.0763 (4)	0.0135 (7)	
H25A	0.342750	0.627499	0.140439	0.016*	
H25B	0.320515	0.610250	−0.027422	0.016*	
C26	0.2515 (6)	0.54938 (14)	0.1015 (4)	0.0152 (7)	
H26A	0.096396	0.557673	0.084466	0.018*	
H26B	0.268389	0.521627	0.030228	0.018*	
C27	0.7347 (7)	0.64190 (15)	0.0996 (5)	0.0230 (9)	
H27A	0.692865	0.655692	−0.001365	0.035*	
H27B	0.890107	0.635485	0.126803	0.035*	
H27C	0.697421	0.667793	0.167558	0.035*	

### Bis(4-methylpiperidine-κN)gold(I) chloride (1) Atomic displacement parameters (Å^2^)

**Table:**

	*U*^11^	*U*^22^	*U*^33^	*U*^12^	*U*^13^	*U*^23^
Au1	0.01176 (6)	0.01306 (6)	0.01248 (7)	−0.00121 (6)	0.00385 (4)	0.00032 (6)
Cl1	0.0163 (4)	0.0183 (5)	0.0256 (5)	−0.0030 (4)	0.0060 (4)	0.0074 (4)
N11	0.0093 (14)	0.0160 (15)	0.0139 (16)	−0.0032 (13)	0.0042 (12)	0.0016 (12)
C12	0.0098 (17)	0.0218 (19)	0.0162 (18)	0.0029 (15)	0.0022 (14)	−0.0013 (15)
C13	0.0143 (19)	0.0125 (17)	0.0146 (18)	0.0033 (14)	0.0009 (14)	0.0003 (14)
C14	0.0140 (18)	0.0106 (16)	0.0150 (17)	0.0010 (14)	0.0035 (14)	−0.0013 (13)
C15	0.0134 (18)	0.0145 (17)	0.0141 (18)	0.0020 (14)	0.0033 (14)	−0.0013 (14)
C16	0.0149 (18)	0.0167 (18)	0.0114 (17)	0.0015 (15)	0.0010 (14)	0.0011 (14)
C17	0.022 (2)	0.019 (2)	0.025 (2)	−0.0019 (17)	0.0065 (17)	−0.0006 (16)
N21	0.0144 (15)	0.0098 (14)	0.0162 (15)	−0.0034 (12)	0.0062 (12)	0.0018 (12)
C22	0.020 (2)	0.0163 (19)	0.0183 (19)	0.0056 (15)	0.0064 (16)	0.0030 (15)
C23	0.0125 (18)	0.0165 (17)	0.0168 (18)	0.0012 (14)	0.0032 (14)	0.0021 (14)
C24	0.0188 (19)	0.0071 (17)	0.0167 (17)	−0.0019 (14)	0.0077 (14)	−0.0024 (13)
C25	0.0154 (18)	0.0157 (17)	0.0089 (16)	−0.0015 (14)	0.0018 (14)	0.0024 (13)
C26	0.0126 (19)	0.0157 (17)	0.0162 (18)	−0.0006 (14)	0.0016 (15)	−0.0011 (15)
C27	0.024 (2)	0.0202 (19)	0.026 (2)	−0.0067 (18)	0.0080 (17)	0.0010 (18)

### Bis(4-methylpiperidine-κN)gold(I) chloride (1) Geometric parameters (Å, º)

**Table:**

Au1—N11	2.051 (3)	C14—H14	1.0000
Au1—N21	2.052 (3)	C15—H15A	0.9900
N11—C12	1.487 (5)	C15—H15B	0.9900
N11—C16	1.499 (5)	C16—H16A	0.9900
C12—C13	1.513 (5)	C16—H16B	0.9900
C13—C14	1.536 (5)	C17—H17A	0.9800
C14—C17	1.526 (5)	C17—H17B	0.9800
C14—C15	1.532 (5)	C17—H17C	0.9800
C15—C16	1.517 (5)	N21—H02	0.92 (3)
N21—C26	1.488 (4)	C22—H22A	0.9900
N21—C22	1.490 (5)	C22—H22B	0.9900
C22—C23	1.524 (5)	C23—H23A	0.9900
C23—C24	1.531 (5)	C23—H23B	0.9900
C24—C25	1.524 (5)	C24—H24	1.0000
C24—C27	1.534 (5)	C25—H25A	0.9900
C25—C26	1.517 (5)	C25—H25B	0.9900
N11—H01	0.90 (3)	C26—H26A	0.9900
C12—H12A	0.9900	C26—H26B	0.9900
C12—H12B	0.9900	C27—H27A	0.9800
C13—H13A	0.9900	C27—H27B	0.9800
C13—H13B	0.9900	C27—H27C	0.9800
			
N11—Au1—N21	179.43 (12)	C15—C16—H16A	109.6
C12—N11—C16	109.5 (3)	N11—C16—H16B	109.6
C12—N11—Au1	113.8 (2)	C15—C16—H16B	109.6
C16—N11—Au1	112.8 (2)	H16A—C16—H16B	108.1
N11—C12—C13	111.2 (3)	C14—C17—H17A	109.5
C12—C13—C14	112.4 (3)	C14—C17—H17B	109.5
C17—C14—C15	111.8 (3)	H17A—C17—H17B	109.5
C17—C14—C13	111.9 (3)	C14—C17—H17C	109.5
C15—C14—C13	109.5 (3)	H17A—C17—H17C	109.5
C16—C15—C14	112.2 (3)	H17B—C17—H17C	109.5
N11—C16—C15	110.3 (3)	C26—N21—H02	109 (2)
C26—N21—C22	109.6 (3)	C22—N21—H02	106 (3)
C26—N21—Au1	112.7 (2)	Au1—N21—H02	106 (3)
C22—N21—Au1	112.8 (2)	N21—C22—H22A	109.7
N21—C22—C23	109.9 (3)	C23—C22—H22A	109.7
C22—C23—C24	112.8 (3)	N21—C22—H22B	109.7
C25—C24—C23	110.5 (3)	C23—C22—H22B	109.7
C25—C24—C27	111.2 (3)	H22A—C22—H22B	108.2
C23—C24—C27	111.4 (3)	C22—C23—H23A	109.0
C26—C25—C24	112.6 (3)	C24—C23—H23A	109.0
N21—C26—C25	110.7 (3)	C22—C23—H23B	109.0
C12—N11—H01	109 (3)	C24—C23—H23B	109.0
C16—N11—H01	109 (3)	H23A—C23—H23B	107.8
Au1—N11—H01	102 (3)	C25—C24—H24	107.9
N11—C12—H12A	109.4	C23—C24—H24	107.9
C13—C12—H12A	109.4	C27—C24—H24	107.9
N11—C12—H12B	109.4	C26—C25—H25A	109.1
C13—C12—H12B	109.4	C24—C25—H25A	109.1
H12A—C12—H12B	108.0	C26—C25—H25B	109.1
C12—C13—H13A	109.1	C24—C25—H25B	109.1
C14—C13—H13A	109.1	H25A—C25—H25B	107.8
C12—C13—H13B	109.1	N21—C26—H26A	109.5
C14—C13—H13B	109.1	C25—C26—H26A	109.5
H13A—C13—H13B	107.9	N21—C26—H26B	109.5
C17—C14—H14	107.8	C25—C26—H26B	109.5
C15—C14—H14	107.8	H26A—C26—H26B	108.1
C13—C14—H14	107.8	C24—C27—H27A	109.5
C16—C15—H15A	109.2	C24—C27—H27B	109.5
C14—C15—H15A	109.2	H27A—C27—H27B	109.5
C16—C15—H15B	109.2	C24—C27—H27C	109.5
C14—C15—H15B	109.2	H27A—C27—H27C	109.5
H15A—C15—H15B	107.9	H27B—C27—H27C	109.5
N11—C16—H16A	109.6		
			
C16—N11—C12—C13	60.1 (4)	C26—N21—C22—C23	61.4 (4)
Au1—N11—C12—C13	−67.1 (3)	Au1—N21—C22—C23	−65.0 (3)
N11—C12—C13—C14	−56.3 (4)	N21—C22—C23—C24	−56.1 (4)
C12—C13—C14—C17	175.6 (3)	C22—C23—C24—C25	49.5 (4)
C12—C13—C14—C15	51.1 (4)	C22—C23—C24—C27	173.6 (3)
C17—C14—C15—C16	−176.5 (3)	C23—C24—C25—C26	−49.2 (4)
C13—C14—C15—C16	−52.0 (4)	C27—C24—C25—C26	−173.4 (3)
C12—N11—C16—C15	−60.6 (4)	C22—N21—C26—C25	−61.6 (4)
Au1—N11—C16—C15	67.2 (3)	Au1—N21—C26—C25	64.9 (3)
C14—C15—C16—N11	57.6 (4)	C24—C25—C26—N21	56.0 (4)

### Bis(4-methylpiperidine-κN)gold(I) chloride (1) Hydrogen-bond geometry (Å, º)

**Table:**

*D*—H···*A*	*D*—H	H···*A*	*D*···*A*	*D*—H···*A*
N21—H02···Cl1	0.92 (3)	2.23 (3)	3.144 (3)	171 (3)
N11—H01···Cl1^i^	0.90 (3)	2.25 (3)	3.140 (3)	170 (4)
C16—H16*A*···Cl1^ii^	0.99	2.85	3.623 (4)	136
C22—H22*B*···Au1^ii^	0.99	2.80	3.724 (4)	155
C23—H23*A*···Au1^iii^	0.99	2.85	3.685 (4)	143

Symmetry codes: (i) −*x*, −*y*+1, −*z*+1; (ii) −*x*+1, −*y*+1, −*z*+1; (iii) *x*+1, *y*, *z*.

### Bis(4-methylpiperidine-κN)gold(I) dichloridoaurate(I) (2) Crystal data

**Table:**

[Au(C_6_H_13_N)_2_][AuCl_2_]	*Z* = 2
*M**_r_* = 663.18	*F*(000) = 608
Triclinic, *P*1	*D*_x_ = 2.607 Mg m^−^^3^
*a* = 9.6998 (7) Å	Mo *K*α radiation, λ = 0.71073 Å
*b* = 9.7001 (8) Å	Cell parameters from 20172 reflections
*c* = 10.7194 (5) Å	θ = 2.4–30.8°
α = 102.218 (6)°	µ = 17.65 mm^−^^1^
β = 101.893 (5)°	*T* = 100 K
γ = 114.695 (8)°	Block, colourless
*V* = 844.89 (11) Å^3^	0.15 × 0.03 × 0.03 mm

### Bis(4-methylpiperidine-κN)gold(I) dichloridoaurate(I) (2) Data collection

**Table:**

Oxford Diffraction Xcalibur, Eos diffractometer	4874 independent reflections
Radiation source: Enhance (Mo) X-ray Source	4690 reflections with *I* > 2σ(*I*)
Detector resolution: 16.1419 pixels mm^-1^	*R*_int_ = 0.080
ω scan	θ_max_ = 30.0°, θ_min_ = 2.5°
Absorption correction: multi-scan (CrysAlisPro; Rigaku OD, 2020)	*h* = −13→13
*T*_min_ = 0.388, *T*_max_ = 1.000	*k* = −13→13
54252 measured reflections	*l* = −15→15

### Bis(4-methylpiperidine-κN)gold(I) dichloridoaurate(I) (2) Refinement

**Table:**

Refinement on *F*^2^	Primary atom site location: structure-invariant direct methods
Least-squares matrix: full	Secondary atom site location: difference Fourier map
*R*[*F*^2^ > 2σ(*F*^2^)] = 0.029	Hydrogen site location: mixed
*wR*(*F*^2^) = 0.064	H atoms treated by a mixture of independent and constrained refinement
*S* = 1.13	*w* = 1/[σ^2^(*F*_o_^2^) + (0.0107*P*)^2^ + 9.0373*P*] where *P* = (*F*_o_^2^ + 2*F*_c_^2^)/3
4874 reflections	(Δ/σ)_max_ < 0.001
174 parameters	Δρ_max_ = 2.02 e Å^−^^3^
1 restraint	Δρ_min_ = −2.52 e Å^−^^3^

### Bis(4-methylpiperidine-κN)gold(I) dichloridoaurate(I) (2) Special details

**Table:**

Geometry. Additional structural data: Torsion angles: -3.13 ( 0.18) N11 - Au1 - Au2 - Cl1 -177.48 ( 0.18) N11 - Au1 - Au2 - Cl2 176.53 ( 0.19) N21 - Au1 - Au2 - Cl1 2.18 ( 0.19) N21 - Au1 - Au2 - Cl2 0.00 ( 0.00) Au2_$1 - Au1 - Au2 - Au1_$1 0.00 ( 0.00) Au2 - Au1 - Au2_$1 - Au1_$1 0.55 ( 0.66) C12 - N11 - N21 - C22 -0.66 ( 0.65) C16 - N11 - N21 - C26 Non-bonded distance: 5.3963 (0.0006) Au1 - Au1_$1 3.5051 (0.0008) Au2 - Au2_S1 Angles in H2Cl2 ring: 88.83 ( 2.82) H01 - Cl1 - H02_$1 88.51 ( 2.89) H01_$1 - Cl2 - H02 91.43 ( 2.06) Cl1 - H01 - Cl2_$1 91.13 ( 2.23) Cl1_$1 - H02 - Cl2

### Bis(4-methylpiperidine-κN)gold(I) dichloridoaurate(I) (2) Fractional atomic coordinates and isotropic or equivalent isotropic displacement parameters (Å^2^)

**Table:**

	*x*	*y*	*z*	*U*_iso_*/*U*_eq_	
Au1	0.34494 (3)	0.35477 (3)	0.15546 (3)	0.01219 (6)	
Au2	0.61479 (3)	0.67852 (3)	0.13899 (3)	0.01717 (7)	
Cl1	0.8285 (2)	0.6453 (3)	0.2297 (2)	0.0231 (4)	
Cl2	0.4149 (2)	0.7390 (2)	0.0684 (2)	0.0198 (4)	
N11	0.5267 (7)	0.3046 (7)	0.2280 (6)	0.0112 (11)	
H01	0.606 (9)	0.357 (10)	0.190 (8)	0.01 (2)*	
C12	0.4725 (9)	0.1288 (9)	0.1908 (8)	0.0160 (14)	
H12A	0.566659	0.111854	0.217324	0.019*	
H12B	0.419603	0.076605	0.091666	0.019*	
C13	0.3549 (9)	0.0532 (9)	0.2624 (8)	0.0162 (14)	
H13A	0.322562	−0.062807	0.239218	0.019*	
H13B	0.257095	0.062502	0.229561	0.019*	
C14	0.4282 (10)	0.1339 (10)	0.4167 (8)	0.0179 (15)	
H14	0.518867	0.112370	0.449831	0.022*	
C15	0.4954 (9)	0.3159 (9)	0.4524 (7)	0.0157 (14)	
H15A	0.405314	0.339488	0.428818	0.019*	
H15B	0.551936	0.368507	0.551193	0.019*	
C16	0.6112 (9)	0.3857 (9)	0.3783 (8)	0.0168 (15)	
H16A	0.650234	0.503004	0.402217	0.020*	
H16B	0.705164	0.368771	0.405836	0.020*	
C17	0.3025 (11)	0.0634 (11)	0.4841 (9)	0.0271 (19)	
H17A	0.352868	0.111307	0.582603	0.041*	
H17B	0.259608	−0.053520	0.457264	0.041*	
H17C	0.214780	0.087344	0.455363	0.041*	
N21	0.1546 (7)	0.3937 (7)	0.0850 (6)	0.0127 (12)	
H02	0.182 (13)	0.453 (12)	0.025 (9)	0.03 (3)*	
C22	0.0007 (9)	0.2393 (10)	0.0106 (8)	0.0196 (15)	
H22A	0.015903	0.171266	−0.062462	0.023*	
H22B	−0.084368	0.262574	−0.031300	0.023*	
C23	−0.0507 (9)	0.1495 (10)	0.1071 (9)	0.0209 (16)	
H23A	0.030961	0.119174	0.143159	0.025*	
H23B	−0.153749	0.049084	0.056799	0.025*	
C24	−0.0713 (9)	0.2496 (10)	0.2247 (8)	0.0194 (16)	
H24	−0.164643	0.265908	0.187621	0.023*	
C25	0.0786 (9)	0.4153 (10)	0.2927 (8)	0.0184 (15)	
H25A	0.057033	0.483991	0.359758	0.022*	
H25B	0.168456	0.402692	0.341894	0.022*	
C26	0.1285 (9)	0.4984 (9)	0.1924 (8)	0.0174 (15)	
H26A	0.043789	0.521809	0.149634	0.021*	
H26B	0.228953	0.601968	0.240362	0.021*	
C27	−0.1075 (11)	0.1642 (12)	0.3275 (10)	0.0276 (19)	
H27A	−0.207149	0.061261	0.283271	0.041*	
H27B	−0.119850	0.231831	0.401554	0.041*	
H27C	−0.018602	0.144513	0.363625	0.041*	

### Bis(4-methylpiperidine-κN)gold(I) dichloridoaurate(I) (2) Atomic displacement parameters (Å^2^)

**Table:**

	*U*^11^	*U*^22^	*U*^33^	*U*^12^	*U*^13^	*U*^23^
Au1	0.01125 (12)	0.01256 (13)	0.01533 (12)	0.00698 (9)	0.00522 (9)	0.00603 (10)
Au2	0.01947 (14)	0.01454 (14)	0.01872 (13)	0.00773 (10)	0.00946 (11)	0.00583 (11)
Cl1	0.0200 (9)	0.0265 (10)	0.0237 (9)	0.0100 (8)	0.0095 (7)	0.0102 (8)
Cl2	0.0241 (9)	0.0173 (9)	0.0241 (9)	0.0119 (7)	0.0132 (8)	0.0091 (7)
N11	0.008 (2)	0.012 (3)	0.011 (3)	0.004 (2)	0.000 (2)	0.001 (2)
C12	0.017 (3)	0.014 (3)	0.020 (4)	0.010 (3)	0.008 (3)	0.005 (3)
C13	0.014 (3)	0.014 (3)	0.020 (4)	0.007 (3)	0.005 (3)	0.005 (3)
C14	0.023 (4)	0.020 (4)	0.018 (4)	0.014 (3)	0.010 (3)	0.010 (3)
C15	0.020 (3)	0.018 (4)	0.014 (3)	0.013 (3)	0.007 (3)	0.007 (3)
C16	0.013 (3)	0.015 (3)	0.020 (4)	0.007 (3)	0.005 (3)	0.004 (3)
C17	0.034 (5)	0.027 (4)	0.028 (4)	0.015 (4)	0.018 (4)	0.016 (4)
N21	0.012 (3)	0.010 (3)	0.012 (3)	0.004 (2)	0.002 (2)	0.003 (2)
C22	0.016 (3)	0.022 (4)	0.016 (3)	0.010 (3)	0.001 (3)	0.001 (3)
C23	0.010 (3)	0.022 (4)	0.029 (4)	0.006 (3)	0.003 (3)	0.013 (3)
C24	0.014 (3)	0.024 (4)	0.028 (4)	0.012 (3)	0.008 (3)	0.014 (3)
C25	0.018 (3)	0.024 (4)	0.019 (4)	0.013 (3)	0.008 (3)	0.010 (3)
C26	0.016 (3)	0.018 (4)	0.022 (4)	0.009 (3)	0.008 (3)	0.008 (3)
C27	0.022 (4)	0.035 (5)	0.037 (5)	0.015 (4)	0.018 (4)	0.023 (4)

### Bis(4-methylpiperidine-κN)gold(I) dichloridoaurate(I) (2) Geometric parameters (Å, º)

**Table:**

Au1—N21	2.062 (7)	C13—H13A	0.9900
Au1—N11	2.064 (6)	C13—H13B	0.9900
Au1—Au2	3.2096 (5)	C14—H14	1.0000
Au1—Au2^i^	3.2252 (4)	C15—H15A	0.9900
Au2—Cl1	2.282 (2)	C15—H15B	0.9900
Au2—Cl2	2.282 (2)	C16—H16A	0.9900
N11—C12	1.490 (9)	C16—H16B	0.9900
N11—C16	1.506 (9)	C17—H17A	0.9800
C12—C13	1.525 (10)	C17—H17B	0.9800
C13—C14	1.535 (11)	C17—H17C	0.9800
C14—C15	1.529 (10)	N21—H02	0.95 (6)
C14—C17	1.533 (11)	C22—H22A	0.9900
C15—C16	1.520 (10)	C22—H22B	0.9900
N21—C22	1.498 (9)	C23—H23A	0.9900
N21—C26	1.504 (10)	C23—H23B	0.9900
C22—C23	1.520 (11)	C24—H24	1.0000
C23—C24	1.523 (12)	C25—H25A	0.9900
C24—C27	1.525 (11)	C25—H25B	0.9900
C24—C25	1.531 (11)	C26—H26A	0.9900
C25—C26	1.516 (11)	C26—H26B	0.9900
N11—H01	0.95 (6)	C27—H27A	0.9800
C12—H12A	0.9900	C27—H27B	0.9800
C12—H12B	0.9900	C27—H27C	0.9800
			
N21—Au1—N11	176.8 (2)	C17—C14—H14	108.5
N21—Au1—Au2	95.61 (17)	C13—C14—H14	108.5
N11—Au1—Au2	87.59 (17)	C16—C15—H15A	109.2
N21—Au1—Au2^i^	87.50 (18)	C14—C15—H15A	109.2
N11—Au1—Au2^i^	94.13 (17)	C16—C15—H15B	109.2
Au2—Au1—Au2^i^	66.011 (12)	C14—C15—H15B	109.2
Cl1—Au2—Cl2	173.42 (7)	H15A—C15—H15B	107.9
Cl1—Au2—Au1	96.01 (5)	N11—C16—H16A	109.8
Cl2—Au2—Au1	87.38 (5)	C15—C16—H16A	109.8
Cl1—Au2—Au1^i^	95.47 (5)	N11—C16—H16B	109.8
Cl2—Au2—Au1^i^	88.25 (5)	C15—C16—H16B	109.8
Au1—Au2—Au1^i^	113.989 (13)	H16A—C16—H16B	108.2
Cl1—Au2—Au2^i^	100.58 (6)	C14—C17—H17A	109.5
Cl2—Au2—Au2^i^	85.99 (5)	C14—C17—H17B	109.5
Au1—Au2—Au2^i^	57.208 (12)	H17A—C17—H17B	109.5
Au1^i^—Au2—Au2^i^	56.781 (11)	C14—C17—H17C	109.5
C12—N11—C16	110.0 (6)	H17A—C17—H17C	109.5
C12—N11—Au1	113.2 (4)	H17B—C17—H17C	109.5
C16—N11—Au1	113.2 (5)	C22—N21—H02	110 (7)
N11—C12—C13	110.0 (6)	C26—N21—H02	105 (7)
C12—C13—C14	112.1 (6)	Au1—N21—H02	106 (7)
C15—C14—C17	111.4 (7)	N21—C22—H22A	109.6
C15—C14—C13	109.5 (6)	C23—C22—H22A	109.6
C17—C14—C13	110.5 (7)	N21—C22—H22B	109.6
C16—C15—C14	112.0 (6)	C23—C22—H22B	109.6
N11—C16—C15	109.5 (6)	H22A—C22—H22B	108.1
C22—N21—C26	109.1 (6)	C22—C23—H23A	109.1
C22—N21—Au1	112.1 (5)	C24—C23—H23A	109.1
C26—N21—Au1	114.3 (5)	C22—C23—H23B	109.1
N21—C22—C23	110.2 (6)	C24—C23—H23B	109.1
C22—C23—C24	112.3 (7)	H23A—C23—H23B	107.9
C23—C24—C27	111.7 (7)	C23—C24—H24	108.0
C23—C24—C25	110.2 (6)	C27—C24—H24	108.0
C27—C24—C25	110.7 (7)	C25—C24—H24	108.0
C26—C25—C24	112.6 (7)	C26—C25—H25A	109.1
N21—C26—C25	110.5 (6)	C24—C25—H25A	109.1
C12—N11—H01	112 (5)	C26—C25—H25B	109.1
C16—N11—H01	104 (5)	C24—C25—H25B	109.1
Au1—N11—H01	104 (5)	H25A—C25—H25B	107.8
N11—C12—H12A	109.7	N21—C26—H26A	109.6
C13—C12—H12A	109.7	C25—C26—H26A	109.6
N11—C12—H12B	109.7	N21—C26—H26B	109.6
C13—C12—H12B	109.7	C25—C26—H26B	109.6
H12A—C12—H12B	108.2	H26A—C26—H26B	108.1
C12—C13—H13A	109.2	C24—C27—H27A	109.5
C14—C13—H13A	109.2	C24—C27—H27B	109.5
C12—C13—H13B	109.2	H27A—C27—H27B	109.5
C14—C13—H13B	109.2	C24—C27—H27C	109.5
H13A—C13—H13B	107.9	H27A—C27—H27C	109.5
C15—C14—H14	108.5	H27B—C27—H27C	109.5
			
C16—N11—C12—C13	60.8 (8)	C26—N21—C22—C23	−61.3 (8)
Au1—N11—C12—C13	−66.9 (7)	Au1—N21—C22—C23	66.3 (7)
N11—C12—C13—C14	−57.1 (8)	N21—C22—C23—C24	57.5 (8)
C12—C13—C14—C15	52.6 (8)	C22—C23—C24—C27	−174.6 (7)
C12—C13—C14—C17	175.7 (7)	C22—C23—C24—C25	−51.2 (9)
C17—C14—C15—C16	−175.9 (7)	C23—C24—C25—C26	50.6 (9)
C13—C14—C15—C16	−53.4 (8)	C27—C24—C25—C26	174.6 (7)
C12—N11—C16—C15	−61.3 (8)	C22—N21—C26—C25	60.7 (8)
Au1—N11—C16—C15	66.4 (7)	Au1—N21—C26—C25	−65.7 (7)
C14—C15—C16—N11	58.1 (8)	C24—C25—C26—N21	−56.1 (9)

Symmetry code: (i) −*x*+1, −*y*+1, −*z*.

### Bis(4-methylpiperidine-κN)gold(I) dichloridoaurate(I) (2) Hydrogen-bond geometry (Å, º)

**Table:**

*D*—H···*A*	*D*—H	H···*A*	*D*···*A*	*D*—H···*A*
N11—H01···Cl1	0.95 (6)	2.59 (7)	3.370 (6)	139 (7)
N11—H01···Cl2^i^	0.95 (6)	2.66 (8)	3.312 (7)	126 (6)
N21—H02···Cl2	0.95 (6)	2.61 (9)	3.316 (7)	131 (8)
N21—H02···Cl1^i^	0.95 (6)	2.66 (9)	3.360 (7)	130 (8)
C13—H13*A*···Cl2^ii^	0.99	2.91	3.640 (8)	131

Symmetry codes: (i) −*x*+1, −*y*+1, −*z*; (ii) *x*, *y*−1, *z*.

### Bis(4-methylpiperidine-κN)gold(I) dibromidoaurate(I) (3) Crystal data

**Table:**

[Au(C_6_H_13_N)_2_][AuBr_2_]	*Z* = 2
*M**_r_* = 752.10	*F*(000) = 680
Triclinic, *P*1	*D*_x_ = 2.876 Mg m^−^^3^
*a* = 9.8461 (6) Å	Mo *K*α radiation, λ = 0.71073 Å
*b* = 9.7728 (4) Å	Cell parameters from 12875 reflections
*c* = 10.9461 (5) Å	θ = 2.6–30.3°
α = 100.136 (4)°	µ = 21.46 mm^−^^1^
β = 103.685 (5)°	*T* = 100 K
γ = 116.287 (5)°	Block, colourless
*V* = 868.59 (9) Å^3^	0.08 × 0.06 × 0.05 mm

### Bis(4-methylpiperidine-κN)gold(I) dibromidoaurate(I) (3) Data collection

**Table:**

Oxford Diffraction Xcalibur, Eos diffractometer	5188 independent reflections
Radiation source: Enhance (Mo) X-ray Source	4466 reflections with *I* > 2σ(*I*)
Detector resolution: 16.1419 pixels mm^-1^	*R*_int_ = 0.050
ω scan	θ_max_ = 31.0°, θ_min_ = 2.5°
Absorption correction: multi-scan (CrysAlisPro; Rigaku OD, 2020)	*h* = −14→14
*T*_min_ = 0.576, *T*_max_ = 1.000	*k* = −14→13
48187 measured reflections	*l* = −15→15

### Bis(4-methylpiperidine-κN)gold(I) dibromidoaurate(I) (3) Refinement

**Table:**

Refinement on *F*^2^	Primary atom site location: structure-invariant direct methods
Least-squares matrix: full	Secondary atom site location: difference Fourier map
*R*[*F*^2^ > 2σ(*F*^2^)] = 0.023	Hydrogen site location: mixed
*wR*(*F*^2^) = 0.041	H atoms treated by a mixture of independent and constrained refinement
*S* = 1.05	*w* = 1/[σ^2^(*F*_o_^2^) + (0.0127*P*)^2^ + 1.3346*P*] where *P* = (*F*_o_^2^ + 2*F*_c_^2^)/3
5188 reflections	(Δ/σ)_max_ = 0.001
173 parameters	Δρ_max_ = 1.02 e Å^−^^3^
1 restraint	Δρ_min_ = −1.15 e Å^−^^3^

### Bis(4-methylpiperidine-κN)gold(I) dibromidoaurate(I) (3) Special details

**Table:**

Geometry. Additional structural data: Non-bonded distance: 5.5563 (0.0004) Au1 - Au1_$1 Torsion angles: -3.06 ( 0.09) N11 - Au1 - Au2 - Br1 -175.21 ( 0.09) N11 - Au1 - Au2 - Br2 175.95 ( 0.08) N21 - Au1 - Au2 - Br1 3.79 ( 0.08) N21 - Au1 - Au2 - Br2 0.00 ( 0.00) Au2_$1 - Au1 - Au2 - Au1_$1 0.00 ( 0.00) Au2 - Au1 - Au2_$1 - Au1_$1 2.08 ( 0.31) C12 - N11 - N21 - C22 0.99 ( 0.32) C16 - N11 - N21 - C26 Operators for generating equivalent atoms: $1 -x+1, -y+1, -z

### Bis(4-methylpiperidine-κN)gold(I) dibromidoaurate(I) (3) Fractional atomic coordinates and isotropic or equivalent isotropic displacement parameters (Å^2^)

**Table:**

	*x*	*y*	*z*	*U*_iso_*/*U*_eq_	
Au1	0.34246 (2)	0.35042 (2)	0.15864 (2)	0.01326 (4)	
Au2	0.62704 (2)	0.68148 (2)	0.13846 (2)	0.01800 (4)	
Br1	0.85675 (5)	0.65879 (5)	0.24231 (4)	0.02139 (8)	
Br2	0.42027 (5)	0.74623 (5)	0.06245 (4)	0.01870 (8)	
N11	0.5261 (4)	0.3083 (4)	0.2396 (3)	0.0146 (6)	
H01	0.597 (5)	0.352 (5)	0.199 (4)	0.031 (13)*	
C12	0.4724 (5)	0.1335 (4)	0.2092 (4)	0.0174 (8)	
H12A	0.568195	0.120983	0.238943	0.021*	
H12B	0.418198	0.078799	0.112095	0.021*	
C13	0.3569 (5)	0.0555 (4)	0.2781 (4)	0.0172 (8)	
H13A	0.326771	−0.059071	0.259395	0.021*	
H13B	0.256719	0.058865	0.241634	0.021*	
C14	0.4302 (5)	0.1390 (5)	0.4283 (4)	0.0187 (8)	
H14	0.522920	0.122592	0.465036	0.022*	
C15	0.4963 (5)	0.3198 (4)	0.4574 (4)	0.0170 (8)	
H15A	0.404566	0.338866	0.430613	0.020*	
H15B	0.554663	0.375071	0.554164	0.020*	
C16	0.6104 (4)	0.3918 (4)	0.3849 (3)	0.0158 (7)	
H16A	0.647748	0.508115	0.404629	0.019*	
H16B	0.706631	0.380388	0.416039	0.019*	
C17	0.3069 (5)	0.0671 (5)	0.4946 (4)	0.0272 (9)	
H17A	0.359292	0.118548	0.591142	0.041*	
H17B	0.265399	−0.049068	0.472803	0.041*	
H17C	0.216830	0.085712	0.462499	0.041*	
N21	0.1493 (4)	0.3816 (4)	0.0822 (3)	0.0151 (6)	
H02	0.171 (6)	0.430 (5)	0.021 (4)	0.033 (13)*	
C22	−0.0056 (5)	0.2246 (5)	0.0142 (4)	0.0194 (8)	
H22A	0.007975	0.154374	−0.053028	0.023*	
H22B	−0.091892	0.243355	−0.032194	0.023*	
C23	−0.0549 (5)	0.1417 (5)	0.1134 (4)	0.0226 (8)	
H23A	0.027452	0.115019	0.153904	0.027*	
H23B	−0.159362	0.039296	0.066307	0.027*	
C24	−0.0728 (5)	0.2453 (5)	0.2231 (4)	0.0207 (8)	
H24	−0.166501	0.258762	0.182547	0.025*	
C25	0.0802 (5)	0.4118 (5)	0.2853 (4)	0.0184 (8)	
H25A	0.061751	0.482870	0.347349	0.022*	
H25B	0.170552	0.401935	0.337243	0.022*	
C26	0.1279 (5)	0.4885 (5)	0.1823 (4)	0.0180 (8)	
H26A	0.042993	0.509416	0.136656	0.022*	
H26B	0.230477	0.593130	0.226770	0.022*	
C27	−0.1067 (5)	0.1650 (6)	0.3281 (5)	0.0303 (10)	
H27A	−0.205630	0.058909	0.286356	0.045*	
H27B	−0.121036	0.232218	0.395758	0.045*	
H27C	−0.015303	0.152108	0.369797	0.045*	

### Bis(4-methylpiperidine-κN)gold(I) dibromidoaurate(I) (3) Atomic displacement parameters (Å^2^)

**Table:**

	*U*^11^	*U*^22^	*U*^33^	*U*^12^	*U*^13^	*U*^23^
Au1	0.01451 (7)	0.01374 (7)	0.01331 (7)	0.00839 (6)	0.00551 (5)	0.00415 (5)
Au2	0.02091 (8)	0.01803 (8)	0.01796 (8)	0.01101 (7)	0.00943 (6)	0.00563 (6)
Br1	0.01983 (19)	0.0256 (2)	0.01905 (19)	0.01164 (17)	0.00751 (16)	0.00668 (16)
Br2	0.0237 (2)	0.01820 (19)	0.01945 (18)	0.01302 (17)	0.01113 (16)	0.00622 (15)
N11	0.0174 (16)	0.0147 (16)	0.0156 (15)	0.0109 (14)	0.0062 (13)	0.0056 (13)
C12	0.0201 (19)	0.017 (2)	0.0172 (18)	0.0114 (17)	0.0070 (16)	0.0030 (15)
C13	0.0181 (19)	0.0107 (18)	0.0208 (19)	0.0073 (16)	0.0052 (16)	0.0029 (15)
C14	0.0190 (19)	0.021 (2)	0.0200 (19)	0.0128 (17)	0.0076 (16)	0.0083 (16)
C15	0.0174 (19)	0.018 (2)	0.0134 (17)	0.0084 (16)	0.0043 (15)	0.0044 (15)
C16	0.0159 (18)	0.0138 (19)	0.0117 (17)	0.0056 (16)	0.0016 (14)	0.0010 (14)
C17	0.033 (2)	0.029 (2)	0.022 (2)	0.014 (2)	0.0141 (19)	0.0124 (19)
N21	0.0174 (16)	0.0162 (17)	0.0123 (15)	0.0092 (14)	0.0055 (13)	0.0038 (13)
C22	0.0160 (19)	0.024 (2)	0.0132 (18)	0.0102 (17)	0.0010 (15)	0.0012 (15)
C23	0.017 (2)	0.018 (2)	0.026 (2)	0.0055 (17)	0.0049 (17)	0.0050 (17)
C24	0.0137 (19)	0.023 (2)	0.025 (2)	0.0086 (17)	0.0069 (16)	0.0090 (17)
C25	0.019 (2)	0.021 (2)	0.0171 (18)	0.0115 (17)	0.0081 (16)	0.0043 (16)
C26	0.020 (2)	0.019 (2)	0.0216 (19)	0.0137 (17)	0.0107 (16)	0.0084 (16)
C27	0.025 (2)	0.038 (3)	0.034 (3)	0.015 (2)	0.015 (2)	0.021 (2)

### Bis(4-methylpiperidine-κN)gold(I) dibromidoaurate(I) (3) Geometric parameters (Å, º)

**Table:**

Au1—N11	2.062 (3)	C13—H13A	0.9900
Au1—N21	2.064 (3)	C13—H13B	0.9900
Au1—Au2	3.2988 (3)	C14—H14	1.0000
Au1—Au2^i^	3.3094 (2)	C15—H15A	0.9900
Au2—Br2	2.4006 (4)	C15—H15B	0.9900
Au2—Br1	2.4027 (4)	C16—H16A	0.9900
N11—C16	1.485 (5)	C16—H16B	0.9900
N11—C12	1.492 (5)	C17—H17A	0.9800
C12—C13	1.511 (5)	C17—H17B	0.9800
C13—C14	1.527 (5)	C17—H17C	0.9800
C14—C15	1.527 (5)	N21—H02	0.89 (3)
C14—C17	1.529 (5)	C22—H22A	0.9900
C15—C16	1.518 (5)	C22—H22B	0.9900
N21—C22	1.491 (5)	C23—H23A	0.9900
N21—C26	1.496 (5)	C23—H23B	0.9900
C22—C23	1.511 (5)	C24—H24	1.0000
C23—C24	1.526 (6)	C25—H25A	0.9900
C24—C27	1.521 (5)	C25—H25B	0.9900
C24—C25	1.528 (5)	C26—H26A	0.9900
C25—C26	1.513 (5)	C26—H26B	0.9900
N11—H01	0.90 (3)	C27—H27A	0.9800
C12—H12A	0.9900	C27—H27B	0.9800
C12—H12B	0.9900	C27—H27C	0.9800
			
N11—Au1—N21	176.30 (13)	C16—C15—H15A	109.1
N11—Au1—Au2	86.25 (9)	C14—C15—H15A	109.1
N21—Au1—Au2	97.32 (9)	C16—C15—H15B	109.1
N11—Au1—Au2^i^	96.43 (9)	C14—C15—H15B	109.1
N21—Au1—Au2^i^	85.98 (8)	H15A—C15—H15B	107.8
Au2—Au1—Au2^i^	65.548 (9)	N11—C16—H16A	109.7
Br2—Au2—Br1	170.628 (15)	C15—C16—H16A	109.7
Br2—Au2—Au1	87.120 (12)	N11—C16—H16B	109.7
Br1—Au2—Au1	98.067 (12)	C15—C16—H16B	109.7
Br2—Au2—Au1^i^	87.536 (11)	H16A—C16—H16B	108.2
Br1—Au2—Au1^i^	97.312 (11)	C14—C17—H17A	109.5
Au1—Au2—Au1^i^	114.452 (8)	C14—C17—H17B	109.5
C16—N11—C12	109.8 (3)	H17A—C17—H17B	109.5
C16—N11—Au1	113.2 (2)	C14—C17—H17C	109.5
C12—N11—Au1	113.4 (2)	H17A—C17—H17C	109.5
N11—C12—C13	110.8 (3)	H17B—C17—H17C	109.5
C12—C13—C14	112.3 (3)	C22—N21—H02	108 (3)
C15—C14—C13	109.3 (3)	C26—N21—H02	107 (3)
C15—C14—C17	111.5 (3)	Au1—N21—H02	106 (3)
C13—C14—C17	111.5 (3)	N21—C22—H22A	109.5
C16—C15—C14	112.4 (3)	C23—C22—H22A	109.5
N11—C16—C15	110.0 (3)	N21—C22—H22B	109.5
C22—N21—C26	109.5 (3)	C23—C22—H22B	109.5
C22—N21—Au1	111.9 (2)	H22A—C22—H22B	108.1
C26—N21—Au1	114.0 (2)	C22—C23—H23A	109.1
N21—C22—C23	110.6 (3)	C24—C23—H23A	109.1
C22—C23—C24	112.6 (3)	C22—C23—H23B	109.1
C27—C24—C23	111.1 (3)	C24—C23—H23B	109.1
C27—C24—C25	110.8 (3)	H23A—C23—H23B	107.8
C23—C24—C25	109.7 (3)	C27—C24—H24	108.4
C26—C25—C24	112.4 (3)	C23—C24—H24	108.4
N21—C26—C25	111.1 (3)	C25—C24—H24	108.4
C16—N11—H01	108 (3)	C26—C25—H25A	109.1
C12—N11—H01	109 (3)	C24—C25—H25A	109.1
Au1—N11—H01	103 (3)	C26—C25—H25B	109.1
N11—C12—H12A	109.5	C24—C25—H25B	109.1
C13—C12—H12A	109.5	H25A—C25—H25B	107.9
N11—C12—H12B	109.5	N21—C26—H26A	109.4
C13—C12—H12B	109.5	C25—C26—H26A	109.4
H12A—C12—H12B	108.1	N21—C26—H26B	109.4
C12—C13—H13A	109.1	C25—C26—H26B	109.4
C14—C13—H13A	109.1	H26A—C26—H26B	108.0
C12—C13—H13B	109.1	C24—C27—H27A	109.5
C14—C13—H13B	109.1	C24—C27—H27B	109.5
H13A—C13—H13B	107.9	H27A—C27—H27B	109.5
C15—C14—H14	108.2	C24—C27—H27C	109.5
C13—C14—H14	108.2	H27A—C27—H27C	109.5
C17—C14—H14	108.2	H27B—C27—H27C	109.5
			
C16—N11—C12—C13	60.2 (4)	C26—N21—C22—C23	−60.0 (4)
Au1—N11—C12—C13	−67.5 (3)	Au1—N21—C22—C23	67.3 (3)
N11—C12—C13—C14	−56.5 (4)	N21—C22—C23—C24	57.3 (4)
C12—C13—C14—C15	51.7 (4)	C22—C23—C24—C27	−174.6 (3)
C12—C13—C14—C17	175.4 (3)	C22—C23—C24—C25	−51.7 (4)
C13—C14—C15—C16	−52.4 (4)	C27—C24—C25—C26	174.0 (3)
C17—C14—C15—C16	−176.1 (3)	C23—C24—C25—C26	51.0 (4)
C12—N11—C16—C15	−60.4 (4)	C22—N21—C26—C25	59.7 (4)
Au1—N11—C16—C15	67.4 (3)	Au1—N21—C26—C25	−66.5 (3)
C14—C15—C16—N11	57.7 (4)	C24—C25—C26—N21	−56.1 (4)

Symmetry code: (i) −*x*+1, −*y*+1, −*z*.

### Bis(4-methylpiperidine-κN)gold(I) dibromidoaurate(I) (3) Hydrogen-bond geometry (Å, º)

**Table:**

*D*—H···*A*	*D*—H	H···*A*	*D*···*A*	*D*—H···*A*
N11—H01···Br2^i^	0.90 (3)	2.80 (4)	3.457 (3)	131 (4)
N11—H01···Br1	0.90 (3)	2.81 (4)	3.518 (3)	136 (4)
N21—H02···Br1^i^	0.89 (3)	2.77 (4)	3.487 (3)	138 (4)
N21—H02···Br2	0.89 (3)	2.84 (4)	3.462 (3)	128 (4)
C13—H13*A*···Br2^ii^	0.99	3.19	3.867 (4)	127

Symmetry codes: (i) −*x*+1, −*y*+1, −*z*; (ii) *x*, *y*−1, *z*.

### Chlorido(4-methylpiperidine-κN)gold(I) bis(4-methylpiperidine-κN)gold(I) chloride dichloromethane monosolvate (4) Crystal data

**Table:**

[Au(C_6_H_13_N)_2_]Cl·[AuCl(C_6_H_13_N)]·CH_2_Cl_2_	*F*(000) = 3216
*M**_r_* = 847.28	*D*_x_ = 2.079 Mg m^−^^3^
Monoclinic, *P*2_1_/*c*	Mo *K*α radiation, λ = 0.71073 Å
*a* = 20.5785 (7) Å	Cell parameters from 14529 reflections
*b* = 16.0876 (4) Å	θ = 2.4–26.3°
*c* = 18.2247 (7) Å	µ = 11.23 mm^−^^1^
β = 116.196 (5)°	*T* = 101 K
*V* = 5413.7 (4) Å^3^	Block, colourless
*Z* = 8	0.25 × 0.1 × 0.1 mm

### Chlorido(4-methylpiperidine-κN)gold(I) bis(4-methylpiperidine-κN)gold(I) chloride dichloromethane monosolvate (4) Data collection

**Table:**

Oxford Diffraction Xcalibur, Eos diffractometer	13423 independent reflections
Radiation source: Enhance (Mo) X-ray Source	11445 reflections with *I* > 2σ(*I*)
Detector resolution: 16.1419 pixels mm^-1^	*R*_int_ = 0.122
ω scan	θ_max_ = 28.3°, θ_min_ = 2.2°
Absorption correction: multi-scan (CrysAlisPro; Rigaku OD, 2020)	*h* = −27→27
*T*_min_ = 0.580, *T*_max_ = 1.000	*k* = −21→21
134776 measured reflections	*l* = −24→24

### Chlorido(4-methylpiperidine-κN)gold(I) bis(4-methylpiperidine-κN)gold(I) chloride dichloromethane monosolvate (4) Refinement

**Table:**

Refinement on *F*^2^	Primary atom site location: structure-invariant direct methods
Least-squares matrix: full	Secondary atom site location: difference Fourier map
*R*[*F*^2^ > 2σ(*F*^2^)] = 0.040	Hydrogen site location: mixed
*wR*(*F*^2^) = 0.070	H atoms treated by a mixture of independent and constrained refinement
*S* = 1.09	*w* = 1/[σ^2^(*F*_o_^2^) + (0.0102*P*)^2^ + 6.0221*P*] where *P* = (*F*_o_^2^ + 2*F*_c_^2^)/3
13423 reflections	(Δ/σ)_max_ = 0.001
530 parameters	Δρ_max_ = 2.02 e Å^−^^3^
15 restraints	Δρ_min_ = −1.91 e Å^−^^3^

### Chlorido(4-methylpiperidine-κN)gold(I) bis(4-methylpiperidine-κN)gold(I) chloride dichloromethane monosolvate (4) Special details

**Table:**

Geometry. Additional structural data: Torsion angles: 85.28 ( 0.32) N11 - Au1 - Au2 - N31 -93.19 ( 0.24) N11 - Au1 - Au2 - Cl1 -93.64 ( 0.31) N21 - Au1 - Au2 - N31 87.89 ( 0.24) N21 - Au1 - Au2 - Cl1 -84.62 ( 0.33) N41 - Au3 - Au4 - N61 95.00 ( 0.24) N41 - Au3 - Au4 - Cl2 93.92 ( 0.33) N51 - Au3 - Au4 - N61 -86.46 ( 0.25) N51 - Au3 - Au4 - Cl2 5.18 ( 0.90) C12 - N11 - N21 - C22 -119.25 ( 0.81) C16 - N11 - N21 - C22 132.02 ( 0.84) C12 - N11 - N21 - C26 132.02 ( 0.84) C12 - N11 - N21 - C26 -4.21 ( 0.93) C42 - N41 - N51 - C52 120.72 ( 0.84) C46 - N41 - N51 - C52 -129.55 ( 0.85) C42 - N41 - N51 - C56 -129.55 ( 0.85) C42 - N41 - N51 - C56 Angles H···Cl···H: 82.86 ( 3.36) H11 - Cl3 - H31 80.32 ( 3.36) H41 - Cl4 - H61

### Chlorido(4-methylpiperidine-κN)gold(I) bis(4-methylpiperidine-κN)gold(I) chloride dichloromethane monosolvate (4) Fractional atomic coordinates and isotropic or equivalent isotropic displacement parameters (Å^2^)

**Table:**

	*x*	*y*	*z*	*U*_iso_*/*U*_eq_	
Au1	−0.00202 (2)	0.49114 (2)	0.16824 (2)	0.01553 (10)	
Au2	0.02294 (2)	0.29093 (2)	0.14623 (2)	0.01759 (10)	
Au3	0.49092 (2)	0.48860 (2)	0.32562 (2)	0.01468 (9)	
Au4	0.51565 (2)	0.29110 (2)	0.36978 (2)	0.01682 (10)	
Cl1	0.04557 (13)	0.26028 (15)	0.27672 (17)	0.0199 (6)	
Cl2	0.54217 (14)	0.25973 (15)	0.26510 (18)	0.0210 (6)	
Cl3	−0.14385 (12)	0.43026 (14)	−0.06278 (14)	0.0176 (5)	
Cl4	0.35323 (12)	0.43684 (14)	0.42016 (14)	0.0171 (5)	
N11	−0.1105 (4)	0.4626 (6)	0.1235 (5)	0.022 (2)	
H11	−0.120 (5)	0.454 (7)	0.076 (3)	0.026*	
C12	−0.1544 (6)	0.5345 (7)	0.1302 (7)	0.027 (3)	
H12A	−0.206637	0.520966	0.101332	0.033*	
H12B	−0.145464	0.584359	0.104040	0.033*	
C13	−0.1337 (5)	0.5525 (6)	0.2192 (7)	0.022 (2)	
H13A	−0.164700	0.597953	0.222449	0.027*	
H13B	−0.082917	0.572198	0.245668	0.027*	
C14	−0.1409 (6)	0.4778 (6)	0.2667 (6)	0.021 (2)	
H14	−0.193596	0.464758	0.245235	0.025*	
C15	−0.1037 (5)	0.4015 (6)	0.2513 (6)	0.019 (2)	
H15A	−0.050539	0.408929	0.280040	0.023*	
H15B	−0.116234	0.351498	0.274063	0.023*	
C16	−0.1262 (5)	0.3883 (6)	0.1614 (7)	0.021 (2)	
H16A	−0.099997	0.339687	0.154181	0.025*	
H16B	−0.178699	0.376047	0.133246	0.025*	
C17	−0.1125 (6)	0.4981 (7)	0.3567 (7)	0.035 (3)	
H17A	−0.060280	0.508489	0.380120	0.052*	
H17B	−0.121780	0.451130	0.385027	0.052*	
H17C	−0.137105	0.547699	0.363137	0.052*	
N21	0.1035 (4)	0.5275 (5)	0.2110 (5)	0.0165 (17)	
H21	0.115 (5)	0.526 (6)	0.174 (4)	0.020*	
C22	0.1166 (6)	0.6129 (6)	0.2472 (6)	0.023 (3)	
H22A	0.081939	0.652411	0.207607	0.027*	
H22B	0.166235	0.631089	0.259354	0.027*	
C23	0.1073 (6)	0.6127 (6)	0.3247 (7)	0.023 (2)	
H23A	0.055927	0.601273	0.310880	0.028*	
H23B	0.119230	0.668603	0.349868	0.028*	
C24	0.1545 (5)	0.5488 (6)	0.3866 (7)	0.020 (2)	
H24	0.206050	0.567176	0.407701	0.024*	
C25	0.1476 (5)	0.4645 (6)	0.3464 (6)	0.017 (2)	
H25A	0.184207	0.426436	0.385755	0.021*	
H25B	0.099169	0.441272	0.333286	0.021*	
C26	0.1574 (5)	0.4675 (6)	0.2695 (7)	0.019 (2)	
H26A	0.149851	0.411470	0.244571	0.022*	
H26B	0.207262	0.485668	0.282373	0.022*	
C27	0.1364 (5)	0.5440 (7)	0.4586 (6)	0.027 (2)	
H27A	0.086038	0.525884	0.439523	0.041*	
H27B	0.142650	0.598881	0.484136	0.041*	
H27C	0.168704	0.504023	0.498717	0.041*	
N31	−0.0004 (4)	0.3160 (5)	0.0252 (5)	0.0177 (19)	
H31	−0.044 (2)	0.322 (6)	0.001 (5)	0.021*	
C32	−0.0067 (5)	0.2378 (6)	−0.0234 (7)	0.020 (2)	
H32A	−0.023819	0.251855	−0.081915	0.024*	
H32B	−0.042413	0.199609	−0.018603	0.024*	
C33	0.0673 (5)	0.1952 (6)	0.0091 (6)	0.023 (2)	
H33A	0.081313	0.176391	0.065813	0.027*	
H33B	0.063100	0.145339	−0.024515	0.027*	
C34	0.1270 (6)	0.2507 (6)	0.0086 (8)	0.020 (3)	
H34	0.115419	0.263182	−0.049648	0.024*	
C35	0.1277 (5)	0.3334 (6)	0.0513 (6)	0.022 (2)	
H35A	0.145567	0.323307	0.110620	0.026*	
H35B	0.161797	0.372206	0.044044	0.026*	
C36	0.0532 (5)	0.3737 (6)	0.0181 (6)	0.023 (2)	
H36A	0.056125	0.425300	0.048931	0.027*	
H36B	0.037044	0.388730	−0.040052	0.027*	
C37	0.2005 (6)	0.2085 (7)	0.0476 (7)	0.032 (3)	
H37A	0.209469	0.187211	0.101577	0.048*	
H37B	0.238254	0.248766	0.053413	0.048*	
H37C	0.201261	0.162363	0.012866	0.048*	
N41	0.3823 (4)	0.4645 (6)	0.2614 (5)	0.0184 (19)	
H41	0.370 (5)	0.457 (7)	0.298 (4)	0.022*	
C42	0.3432 (6)	0.5369 (7)	0.2122 (7)	0.027 (3)	
H42A	0.354037	0.586613	0.247699	0.032*	
H42B	0.290341	0.526656	0.188180	0.032*	
C43	0.3652 (6)	0.5527 (6)	0.1447 (6)	0.025 (3)	
H43A	0.416765	0.569628	0.169228	0.030*	
H43B	0.336110	0.599171	0.110332	0.030*	
C44	0.3551 (5)	0.4764 (6)	0.0906 (6)	0.020 (2)	
H44	0.301876	0.466137	0.059446	0.024*	
C45	0.3880 (6)	0.4011 (6)	0.1415 (7)	0.022 (2)	
H45A	0.441445	0.405876	0.166272	0.026*	
H45B	0.373881	0.351296	0.105944	0.026*	
C46	0.3642 (6)	0.3904 (7)	0.2087 (7)	0.030 (3)	
H46A	0.311222	0.380915	0.184035	0.036*	
H46B	0.388377	0.341123	0.242155	0.036*	
C47	0.3849 (6)	0.4921 (7)	0.0284 (6)	0.032 (3)	
H47A	0.436949	0.503463	0.057320	0.048*	
H47B	0.360123	0.540071	−0.005628	0.048*	
H47C	0.376662	0.442945	−0.006287	0.048*	
N51	0.5979 (5)	0.5227 (5)	0.3903 (5)	0.0198 (19)	
H51	0.607 (5)	0.524 (6)	0.438 (3)	0.024*	
C52	0.6127 (6)	0.6070 (6)	0.3682 (7)	0.025 (3)	
H52A	0.662321	0.624190	0.406880	0.030*	
H52B	0.578269	0.647171	0.372847	0.030*	
C53	0.6055 (5)	0.6089 (6)	0.2811 (7)	0.023 (3)	
H53A	0.554437	0.598064	0.242136	0.028*	
H53B	0.618337	0.665074	0.269491	0.028*	
C54	0.6537 (5)	0.5451 (6)	0.2678 (7)	0.022 (2)	
H54	0.705285	0.562158	0.300923	0.026*	
C55	0.6428 (5)	0.4593 (6)	0.2985 (6)	0.018 (2)	
H55A	0.594170	0.437916	0.261311	0.022*	
H55B	0.679171	0.419995	0.296918	0.022*	
C56	0.6497 (5)	0.4630 (7)	0.3839 (7)	0.024 (3)	
H56A	0.640831	0.407025	0.400193	0.029*	
H56B	0.699679	0.479645	0.421893	0.029*	
C57	0.6394 (6)	0.5406 (7)	0.1787 (6)	0.031 (3)	
H57A	0.589309	0.523066	0.145183	0.046*	
H57B	0.672562	0.500343	0.172767	0.046*	
H57C	0.647231	0.595475	0.160586	0.046*	
N61	0.4912 (4)	0.3156 (5)	0.4659 (6)	0.022 (2)	
H61	0.449 (2)	0.330 (6)	0.445 (6)	0.026*	
C62	0.4842 (6)	0.2387 (6)	0.5079 (8)	0.027 (3)	
H62A	0.448181	0.200933	0.467391	0.033*	
H62B	0.466938	0.253585	0.548994	0.033*	
C63	0.5584 (6)	0.1942 (6)	0.5504 (7)	0.029 (3)	
H63A	0.553716	0.144733	0.580026	0.035*	
H63B	0.573431	0.175051	0.508530	0.035*	
C64	0.6159 (6)	0.2519 (6)	0.6101 (8)	0.026 (3)	
H64	0.604230	0.263581	0.656895	0.031*	
C65	0.6170 (5)	0.3349 (6)	0.5682 (6)	0.019 (2)	
H65A	0.650288	0.374174	0.609564	0.023*	
H65B	0.635429	0.325182	0.527126	0.023*	
C66	0.5420 (5)	0.3729 (5)	0.5268 (6)	0.017 (2)	
H66A	0.524759	0.385862	0.568291	0.020*	
H66B	0.544164	0.425494	0.499714	0.020*	
C67	0.6890 (6)	0.2084 (7)	0.6427 (7)	0.036 (3)	
H67A	0.688606	0.159580	0.674544	0.054*	
H67B	0.727128	0.246804	0.677648	0.054*	
H67C	0.698462	0.190978	0.596735	0.054*	
C1	0.2418 (5)	0.2663 (7)	0.3577 (7)	0.026 (3)	
H1A	0.261404	0.321997	0.379147	0.031*	
H1B	0.189596	0.266190	0.343926	0.031*	
Cl5	0.25277 (16)	0.24644 (16)	0.2668 (2)	0.0367 (8)	
Cl6	0.28565 (14)	0.19267 (16)	0.43223 (16)	0.0308 (6)	
C2	0.7414 (6)	0.2375 (6)	0.3816 (8)	0.024 (3)	
H2A	0.690937	0.247144	0.340229	0.029*	
H2B	0.753370	0.178499	0.377869	0.029*	
Cl7	0.74773 (16)	0.25645 (18)	0.4784 (2)	0.0346 (8)	
Cl8	0.80057 (14)	0.30207 (16)	0.35950 (16)	0.0285 (6)	

### Chlorido(4-methylpiperidine-κN)gold(I) bis(4-methylpiperidine-κN)gold(I) chloride dichloromethane monosolvate (4) Atomic displacement parameters (Å^2^)

**Table:**

	*U*^11^	*U*^22^	*U*^33^	*U*^12^	*U*^13^	*U*^23^
Au1	0.01286 (18)	0.0209 (2)	0.0121 (2)	0.00020 (14)	0.00482 (15)	0.00123 (15)
Au2	0.01393 (18)	0.0174 (2)	0.0192 (2)	0.00127 (14)	0.00528 (16)	0.00212 (16)
Au3	0.01280 (17)	0.0194 (2)	0.0115 (2)	0.00030 (14)	0.00511 (15)	0.00025 (15)
Au4	0.01545 (19)	0.0153 (2)	0.0196 (2)	0.00096 (14)	0.00757 (15)	−0.00137 (15)
Cl1	0.0172 (12)	0.0207 (12)	0.0178 (15)	−0.0011 (9)	0.0040 (11)	0.0016 (10)
Cl2	0.0221 (13)	0.0219 (13)	0.0203 (15)	0.0032 (9)	0.0105 (12)	−0.0024 (10)
Cl3	0.0168 (12)	0.0178 (11)	0.0171 (12)	−0.0010 (9)	0.0066 (10)	0.0009 (10)
Cl4	0.0145 (12)	0.0192 (12)	0.0173 (12)	−0.0008 (9)	0.0068 (10)	−0.0017 (9)
N11	0.013 (4)	0.039 (5)	0.010 (4)	0.000 (4)	0.001 (4)	−0.004 (4)
C12	0.014 (5)	0.039 (7)	0.023 (6)	0.008 (4)	0.002 (5)	0.009 (5)
C13	0.012 (5)	0.021 (6)	0.031 (7)	0.002 (4)	0.007 (5)	−0.001 (5)
C14	0.024 (5)	0.027 (6)	0.012 (5)	−0.008 (4)	0.007 (5)	−0.004 (4)
C15	0.021 (5)	0.018 (5)	0.019 (6)	−0.005 (4)	0.010 (5)	−0.004 (4)
C16	0.015 (5)	0.014 (5)	0.037 (7)	−0.008 (4)	0.014 (5)	−0.012 (5)
C17	0.046 (7)	0.030 (6)	0.028 (7)	−0.005 (5)	0.017 (6)	0.003 (5)
N21	0.011 (4)	0.031 (5)	0.009 (4)	0.001 (3)	0.007 (3)	0.001 (4)
C22	0.026 (6)	0.020 (6)	0.012 (6)	−0.006 (4)	−0.002 (5)	−0.001 (4)
C23	0.027 (6)	0.013 (5)	0.027 (6)	0.004 (4)	0.009 (5)	−0.006 (4)
C24	0.013 (5)	0.024 (6)	0.023 (6)	−0.001 (4)	0.008 (5)	−0.005 (5)
C25	0.020 (5)	0.010 (5)	0.021 (6)	0.002 (4)	0.009 (5)	0.000 (4)
C26	0.015 (5)	0.016 (5)	0.022 (6)	0.005 (4)	0.006 (4)	−0.003 (4)
C27	0.022 (6)	0.038 (6)	0.016 (6)	−0.006 (4)	0.004 (5)	−0.001 (5)
N31	0.016 (4)	0.008 (4)	0.018 (5)	−0.001 (3)	−0.002 (4)	0.002 (4)
C32	0.022 (5)	0.020 (5)	0.018 (6)	−0.003 (4)	0.008 (5)	0.004 (5)
C33	0.034 (6)	0.013 (5)	0.018 (5)	0.007 (4)	0.008 (5)	0.001 (4)
C34	0.020 (5)	0.029 (7)	0.013 (6)	0.002 (4)	0.009 (5)	−0.001 (4)
C35	0.012 (5)	0.028 (6)	0.021 (6)	0.001 (4)	0.004 (4)	0.009 (5)
C36	0.022 (5)	0.016 (5)	0.019 (6)	−0.003 (4)	−0.001 (4)	0.009 (4)
C37	0.028 (6)	0.036 (7)	0.034 (7)	0.012 (5)	0.015 (5)	−0.009 (5)
N41	0.012 (4)	0.033 (5)	0.009 (4)	0.000 (3)	0.004 (4)	0.000 (4)
C42	0.021 (6)	0.034 (7)	0.024 (6)	0.012 (5)	0.008 (5)	−0.001 (5)
C43	0.031 (6)	0.018 (6)	0.020 (6)	0.011 (4)	0.006 (5)	0.000 (5)
C44	0.017 (5)	0.022 (5)	0.013 (5)	−0.004 (4)	−0.002 (4)	0.008 (4)
C45	0.030 (6)	0.014 (5)	0.016 (6)	0.002 (4)	0.006 (5)	−0.004 (4)
C46	0.021 (6)	0.031 (6)	0.029 (7)	−0.011 (5)	0.001 (5)	0.004 (5)
C47	0.043 (7)	0.031 (6)	0.013 (6)	−0.008 (5)	0.004 (5)	0.000 (5)
N51	0.024 (5)	0.016 (4)	0.020 (5)	−0.007 (3)	0.010 (4)	0.000 (4)
C52	0.035 (6)	0.015 (5)	0.029 (7)	−0.010 (4)	0.018 (5)	−0.006 (5)
C53	0.021 (6)	0.016 (5)	0.035 (7)	−0.003 (4)	0.014 (5)	−0.005 (5)
C54	0.018 (5)	0.018 (5)	0.028 (6)	0.001 (4)	0.010 (5)	0.008 (5)
C55	0.019 (5)	0.012 (5)	0.028 (6)	0.000 (4)	0.015 (5)	−0.003 (4)
C56	0.010 (5)	0.032 (6)	0.028 (7)	−0.001 (4)	0.006 (5)	0.011 (5)
C57	0.045 (7)	0.026 (6)	0.025 (7)	−0.003 (5)	0.018 (6)	0.002 (5)
N61	0.021 (5)	0.023 (5)	0.023 (5)	−0.002 (4)	0.011 (4)	−0.002 (4)
C62	0.033 (6)	0.018 (6)	0.048 (8)	−0.007 (5)	0.032 (6)	−0.005 (5)
C63	0.036 (7)	0.025 (6)	0.032 (6)	0.005 (5)	0.020 (6)	0.007 (5)
C64	0.026 (6)	0.030 (7)	0.026 (7)	0.003 (4)	0.017 (6)	0.001 (5)
C65	0.024 (5)	0.025 (5)	0.015 (5)	−0.010 (4)	0.016 (4)	−0.006 (4)
C66	0.017 (5)	0.006 (4)	0.025 (6)	0.000 (3)	0.008 (4)	−0.001 (4)
C67	0.034 (7)	0.040 (7)	0.029 (6)	0.008 (5)	0.009 (5)	0.017 (6)
C1	0.021 (6)	0.029 (6)	0.026 (7)	0.001 (4)	0.009 (5)	−0.005 (5)
Cl5	0.046 (2)	0.037 (2)	0.028 (2)	0.0022 (13)	0.0183 (17)	0.0111 (12)
Cl6	0.0321 (15)	0.0336 (15)	0.0241 (15)	0.0045 (11)	0.0099 (12)	0.0058 (12)
C2	0.016 (5)	0.021 (5)	0.032 (7)	−0.001 (4)	0.007 (5)	0.001 (5)
Cl7	0.0447 (19)	0.0321 (18)	0.037 (2)	0.0010 (12)	0.0266 (17)	−0.0001 (14)
Cl8	0.0232 (14)	0.0354 (15)	0.0258 (14)	−0.0068 (11)	0.0098 (11)	0.0026 (12)

### Chlorido(4-methylpiperidine-κN)gold(I) bis(4-methylpiperidine-κN)gold(I) chloride dichloromethane monosolvate (4) Geometric parameters (Å, º)

**Table:**

Au1—N21	2.042 (8)	C37—H37C	0.9800
Au1—N11	2.061 (8)	N41—C46	1.471 (13)
Au1—Au2	3.3138 (6)	N41—C42	1.475 (13)
Au2—N31	2.078 (9)	N41—H41	0.81 (3)
Au2—Cl1	2.267 (3)	C42—C43	1.508 (14)
Au3—N41	2.051 (8)	C42—H42A	0.9900
Au3—N51	2.060 (9)	C42—H42B	0.9900
Au3—Au4	3.2619 (5)	C43—C44	1.529 (13)
Au4—N61	2.062 (9)	C43—H43A	0.9900
Au4—Cl2	2.261 (3)	C43—H43B	0.9900
N11—C16	1.486 (13)	C44—C45	1.493 (13)
N11—C12	1.507 (14)	C44—C47	1.530 (14)
N11—H11	0.82 (3)	C44—H44	1.0000
C12—C13	1.512 (14)	C45—C46	1.519 (15)
C12—H12A	0.9900	C45—H45A	0.9900
C12—H12B	0.9900	C45—H45B	0.9900
C13—C14	1.526 (13)	C46—H46A	0.9900
C13—H13A	0.9900	C46—H46B	0.9900
C13—H13B	0.9900	C47—H47A	0.9800
C14—C17	1.513 (14)	C47—H47B	0.9800
C14—C15	1.536 (14)	C47—H47C	0.9800
C14—H14	1.0000	N51—C56	1.478 (13)
C15—C16	1.509 (14)	N51—C52	1.486 (12)
C15—H15A	0.9900	N51—H51	0.81 (3)
C15—H15B	0.9900	C52—C53	1.527 (15)
C16—H16A	0.9900	C52—H52A	0.9900
C16—H16B	0.9900	C52—H52B	0.9900
C17—H17A	0.9800	C53—C54	1.520 (14)
C17—H17B	0.9800	C53—H53A	0.9900
C17—H17C	0.9800	C53—H53B	0.9900
N21—C22	1.497 (12)	C54—C57	1.519 (14)
N21—C26	1.502 (12)	C54—C55	1.543 (13)
N21—H21	0.81 (3)	C54—H54	1.0000
C22—C23	1.506 (14)	C55—C56	1.500 (14)
C22—H22A	0.9900	C55—H55A	0.9900
C22—H22B	0.9900	C55—H55B	0.9900
C23—C24	1.519 (14)	C56—H56A	0.9900
C23—H23A	0.9900	C56—H56B	0.9900
C23—H23B	0.9900	C57—H57A	0.9800
C24—C27	1.516 (14)	C57—H57B	0.9800
C24—C25	1.518 (13)	C57—H57C	0.9800
C24—H24	1.0000	N61—C66	1.467 (12)
C25—C26	1.501 (14)	N61—C62	1.494 (13)
C25—H25A	0.9900	N61—H61	0.81 (3)
C25—H25B	0.9900	C62—C63	1.550 (14)
C26—H26A	0.9900	C62—H62A	0.9900
C26—H26B	0.9900	C62—H62B	0.9900
C27—H27A	0.9800	C63—C64	1.520 (15)
C27—H27B	0.9800	C63—H63A	0.9900
C27—H27C	0.9800	C63—H63B	0.9900
N31—C36	1.492 (12)	C64—C67	1.523 (14)
N31—C32	1.512 (13)	C64—C65	1.543 (13)
N31—H31	0.82 (3)	C64—H64	1.0000
C32—C33	1.531 (13)	C65—C66	1.515 (12)
C32—H32A	0.9900	C65—H65A	0.9900
C32—H32B	0.9900	C65—H65B	0.9900
C33—C34	1.523 (14)	C66—H66A	0.9900
C33—H33A	0.9900	C66—H66B	0.9900
C33—H33B	0.9900	C67—H67A	0.9800
C34—C37	1.518 (13)	C67—H67B	0.9800
C34—C35	1.538 (13)	C67—H67C	0.9800
C34—H34	1.0000	C1—Cl6	1.726 (11)
C35—C36	1.522 (12)	C1—Cl5	1.796 (13)
C35—H35A	0.9900	C1—H1A	0.9900
C35—H35B	0.9900	C1—H1B	0.9900
C36—H36A	0.9900	C2—Cl7	1.738 (13)
C36—H36B	0.9900	C2—Cl8	1.777 (11)
C37—H37A	0.9800	C2—H2A	0.9900
C37—H37B	0.9800	C2—H2B	0.9900
			
N21—Au1—N11	176.2 (4)	C46—N41—C42	109.3 (8)
N21—Au1—Au2	97.4 (2)	C46—N41—Au3	115.1 (6)
N11—Au1—Au2	86.2 (3)	C42—N41—Au3	111.3 (7)
N31—Au2—Cl1	178.1 (2)	C46—N41—H41	109 (8)
N31—Au2—Au1	88.0 (2)	C42—N41—H41	110 (8)
Cl1—Au2—Au1	93.18 (6)	Au3—N41—H41	102 (7)
N41—Au3—N51	175.4 (3)	N41—C42—C43	110.4 (9)
N41—Au3—Au4	88.2 (3)	N41—C42—H42A	109.6
N51—Au3—Au4	96.1 (2)	C43—C42—H42A	109.6
N61—Au4—Cl2	178.1 (3)	N41—C42—H42B	109.6
N61—Au4—Au3	87.3 (2)	C43—C42—H42B	109.6
Cl2—Au4—Au3	94.52 (6)	H42A—C42—H42B	108.1
C16—N11—C12	108.6 (8)	C42—C43—C44	112.7 (9)
C16—N11—Au1	114.5 (6)	C42—C43—H43A	109.1
C12—N11—Au1	112.4 (7)	C44—C43—H43A	109.1
C16—N11—H11	111 (8)	C42—C43—H43B	109.1
C12—N11—H11	111 (8)	C44—C43—H43B	109.1
Au1—N11—H11	100 (7)	H43A—C43—H43B	107.8
N11—C12—C13	109.8 (8)	C45—C44—C43	110.8 (8)
N11—C12—H12A	109.7	C45—C44—C47	111.9 (9)
C13—C12—H12A	109.7	C43—C44—C47	111.1 (8)
N11—C12—H12B	109.7	C45—C44—H44	107.6
C13—C12—H12B	109.7	C43—C44—H44	107.6
H12A—C12—H12B	108.2	C47—C44—H44	107.6
C12—C13—C14	114.0 (9)	C44—C45—C46	111.9 (9)
C12—C13—H13A	108.7	C44—C45—H45A	109.2
C14—C13—H13A	108.7	C46—C45—H45A	109.2
C12—C13—H13B	108.7	C44—C45—H45B	109.2
C14—C13—H13B	108.7	C46—C45—H45B	109.2
H13A—C13—H13B	107.6	H45A—C45—H45B	107.9
C17—C14—C13	111.0 (8)	N41—C46—C45	110.8 (8)
C17—C14—C15	112.7 (9)	N41—C46—H46A	109.5
C13—C14—C15	110.1 (8)	C45—C46—H46A	109.5
C17—C14—H14	107.6	N41—C46—H46B	109.5
C13—C14—H14	107.6	C45—C46—H46B	109.5
C15—C14—H14	107.6	H46A—C46—H46B	108.1
C16—C15—C14	111.9 (9)	C44—C47—H47A	109.5
C16—C15—H15A	109.2	C44—C47—H47B	109.5
C14—C15—H15A	109.2	H47A—C47—H47B	109.5
C16—C15—H15B	109.2	C44—C47—H47C	109.5
C14—C15—H15B	109.2	H47A—C47—H47C	109.5
H15A—C15—H15B	107.9	H47B—C47—H47C	109.5
N11—C16—C15	111.5 (8)	C56—N51—C52	109.3 (8)
N11—C16—H16A	109.3	C56—N51—Au3	114.1 (6)
C15—C16—H16A	109.3	C52—N51—Au3	112.5 (7)
N11—C16—H16B	109.3	C56—N51—H51	106 (8)
C15—C16—H16B	109.3	C52—N51—H51	107 (8)
H16A—C16—H16B	108.0	Au3—N51—H51	108 (8)
C14—C17—H17A	109.5	N51—C52—C53	111.3 (9)
C14—C17—H17B	109.5	N51—C52—H52A	109.4
H17A—C17—H17B	109.5	C53—C52—H52A	109.4
C14—C17—H17C	109.5	N51—C52—H52B	109.4
H17A—C17—H17C	109.5	C53—C52—H52B	109.4
H17B—C17—H17C	109.5	H52A—C52—H52B	108.0
C22—N21—C26	110.1 (8)	C54—C53—C52	112.4 (9)
C22—N21—Au1	112.6 (6)	C54—C53—H53A	109.1
C26—N21—Au1	114.5 (6)	C52—C53—H53A	109.1
C22—N21—H21	110 (7)	C54—C53—H53B	109.1
C26—N21—H21	100 (7)	C52—C53—H53B	109.1
Au1—N21—H21	109 (7)	H53A—C53—H53B	107.8
N21—C22—C23	109.9 (8)	C57—C54—C53	111.8 (9)
N21—C22—H22A	109.7	C57—C54—C55	110.8 (9)
C23—C22—H22A	109.7	C53—C54—C55	109.6 (8)
N21—C22—H22B	109.7	C57—C54—H54	108.2
C23—C22—H22B	109.7	C53—C54—H54	108.2
H22A—C22—H22B	108.2	C55—C54—H54	108.2
C22—C23—C24	113.2 (9)	C56—C55—C54	112.3 (9)
C22—C23—H23A	108.9	C56—C55—H55A	109.2
C24—C23—H23A	108.9	C54—C55—H55A	109.2
C22—C23—H23B	108.9	C56—C55—H55B	109.2
C24—C23—H23B	108.9	C54—C55—H55B	109.2
H23A—C23—H23B	107.8	H55A—C55—H55B	107.9
C27—C24—C25	111.5 (9)	N51—C56—C55	111.5 (9)
C27—C24—C23	110.9 (9)	N51—C56—H56A	109.3
C25—C24—C23	110.7 (9)	C55—C56—H56A	109.3
C27—C24—H24	107.9	N51—C56—H56B	109.3
C25—C24—H24	107.9	C55—C56—H56B	109.3
C23—C24—H24	107.9	H56A—C56—H56B	108.0
C26—C25—C24	113.6 (9)	C54—C57—H57A	109.5
C26—C25—H25A	108.9	C54—C57—H57B	109.5
C24—C25—H25A	108.9	H57A—C57—H57B	109.5
C26—C25—H25B	108.9	C54—C57—H57C	109.5
C24—C25—H25B	108.9	H57A—C57—H57C	109.5
H25A—C25—H25B	107.7	H57B—C57—H57C	109.5
C25—C26—N21	108.8 (8)	C66—N61—C62	109.3 (8)
C25—C26—H26A	109.9	C66—N61—Au4	113.8 (6)
N21—C26—H26A	109.9	C62—N61—Au4	113.1 (7)
C25—C26—H26B	109.9	C66—N61—H61	116 (7)
N21—C26—H26B	109.9	C62—N61—H61	99 (7)
H26A—C26—H26B	108.3	Au4—N61—H61	105 (8)
C24—C27—H27A	109.5	N61—C62—C63	110.0 (8)
C24—C27—H27B	109.5	N61—C62—H62A	109.7
H27A—C27—H27B	109.5	C63—C62—H62A	109.7
C24—C27—H27C	109.5	N61—C62—H62B	109.7
H27A—C27—H27C	109.5	C63—C62—H62B	109.7
H27B—C27—H27C	109.5	H62A—C62—H62B	108.2
C36—N31—C32	110.0 (8)	C64—C63—C62	111.0 (9)
C36—N31—Au2	112.3 (6)	C64—C63—H63A	109.4
C32—N31—Au2	112.4 (6)	C62—C63—H63A	109.4
C36—N31—H31	124 (7)	C64—C63—H63B	109.4
C32—N31—H31	90 (7)	C62—C63—H63B	109.4
Au2—N31—H31	106 (7)	H63A—C63—H63B	108.0
N31—C32—C33	109.4 (8)	C63—C64—C67	108.7 (9)
N31—C32—H32A	109.8	C63—C64—C65	110.6 (10)
C33—C32—H32A	109.8	C67—C64—C65	111.2 (9)
N31—C32—H32B	109.8	C63—C64—H64	108.8
C33—C32—H32B	109.8	C67—C64—H64	108.8
H32A—C32—H32B	108.2	C65—C64—H64	108.8
C34—C33—C32	113.8 (8)	C66—C65—C64	111.2 (8)
C34—C33—H33A	108.8	C66—C65—H65A	109.4
C32—C33—H33A	108.8	C64—C65—H65A	109.4
C34—C33—H33B	108.8	C66—C65—H65B	109.4
C32—C33—H33B	108.8	C64—C65—H65B	109.4
H33A—C33—H33B	107.7	H65A—C65—H65B	108.0
C37—C34—C33	112.0 (9)	N61—C66—C65	110.6 (7)
C37—C34—C35	111.4 (9)	N61—C66—H66A	109.5
C33—C34—C35	109.4 (9)	C65—C66—H66A	109.5
C37—C34—H34	108.0	N61—C66—H66B	109.5
C33—C34—H34	108.0	C65—C66—H66B	109.5
C35—C34—H34	108.0	H66A—C66—H66B	108.1
C36—C35—C34	112.6 (8)	C64—C67—H67A	109.5
C36—C35—H35A	109.1	C64—C67—H67B	109.5
C34—C35—H35A	109.1	H67A—C67—H67B	109.5
C36—C35—H35B	109.1	C64—C67—H67C	109.5
C34—C35—H35B	109.1	H67A—C67—H67C	109.5
H35A—C35—H35B	107.8	H67B—C67—H67C	109.5
N31—C36—C35	110.8 (7)	Cl6—C1—Cl5	111.7 (6)
N31—C36—H36A	109.5	Cl6—C1—H1A	109.3
C35—C36—H36A	109.5	Cl5—C1—H1A	109.3
N31—C36—H36B	109.5	Cl6—C1—H1B	109.3
C35—C36—H36B	109.5	Cl5—C1—H1B	109.3
H36A—C36—H36B	108.1	H1A—C1—H1B	107.9
C34—C37—H37A	109.5	Cl7—C2—Cl8	112.1 (6)
C34—C37—H37B	109.5	Cl7—C2—H2A	109.2
H37A—C37—H37B	109.5	Cl8—C2—H2A	109.2
C34—C37—H37C	109.5	Cl7—C2—H2B	109.2
H37A—C37—H37C	109.5	Cl8—C2—H2B	109.2
H37B—C37—H37C	109.5	H2A—C2—H2B	107.9
			
C16—N11—C12—C13	60.7 (11)	C46—N41—C42—C43	−60.9 (11)
Au1—N11—C12—C13	−67.1 (10)	Au3—N41—C42—C43	67.3 (10)
N11—C12—C13—C14	−55.7 (11)	N41—C42—C43—C44	55.2 (12)
C12—C13—C14—C17	174.6 (9)	C42—C43—C44—C45	−49.4 (12)
C12—C13—C14—C15	49.1 (11)	C42—C43—C44—C47	−174.5 (9)
C17—C14—C15—C16	−173.3 (8)	C43—C44—C45—C46	49.7 (12)
C13—C14—C15—C16	−48.8 (11)	C47—C44—C45—C46	174.3 (8)
C12—N11—C16—C15	−62.6 (10)	C42—N41—C46—C45	62.0 (11)
Au1—N11—C16—C15	63.9 (9)	Au3—N41—C46—C45	−64.0 (10)
C14—C15—C16—N11	57.3 (11)	C44—C45—C46—N41	−57.2 (11)
C26—N21—C22—C23	−61.3 (10)	C56—N51—C52—C53	59.5 (11)
Au1—N21—C22—C23	67.9 (9)	Au3—N51—C52—C53	−68.4 (10)
N21—C22—C23—C24	55.0 (11)	N51—C52—C53—C54	−55.7 (12)
C22—C23—C24—C27	−173.0 (9)	C52—C53—C54—C57	173.2 (9)
C22—C23—C24—C25	−48.8 (12)	C52—C53—C54—C55	50.0 (12)
C27—C24—C25—C26	174.0 (8)	C57—C54—C55—C56	−174.7 (9)
C23—C24—C25—C26	50.1 (11)	C53—C54—C55—C56	−50.9 (11)
C24—C25—C26—N21	−56.7 (11)	C52—N51—C56—C55	−60.7 (11)
C22—N21—C26—C25	61.7 (10)	Au3—N51—C56—C55	66.3 (10)
Au1—N21—C26—C25	−66.4 (9)	C54—C55—C56—N51	57.3 (11)
C36—N31—C32—C33	59.5 (10)	C66—N61—C62—C63	−62.0 (11)
Au2—N31—C32—C33	−66.5 (9)	Au4—N61—C62—C63	65.8 (10)
N31—C32—C33—C34	−56.3 (12)	N61—C62—C63—C64	56.7 (13)
C32—C33—C34—C37	175.4 (9)	C62—C63—C64—C67	−173.6 (10)
C32—C33—C34—C35	51.4 (12)	C62—C63—C64—C65	−51.3 (13)
C37—C34—C35—C36	−175.3 (9)	C63—C64—C65—C66	52.1 (12)
C33—C34—C35—C36	−50.9 (12)	C67—C64—C65—C66	173.0 (9)
C32—N31—C36—C35	−60.6 (10)	C62—N61—C66—C65	63.2 (10)
Au2—N31—C36—C35	65.4 (9)	Au4—N61—C66—C65	−64.3 (9)
C34—C35—C36—N31	56.8 (11)	C64—C65—C66—N61	−58.3 (11)

### Chlorido(4-methylpiperidine-κN)gold(I) bis(4-methylpiperidine-κN)gold(I) chloride dichloromethane monosolvate (4) Hydrogen-bond geometry (Å, º)

**Table:**

*D*—H···*A*	*D*—H	H···*A*	*D*···*A*	*D*—H···*A*
N11—H11···Cl3	0.82 (3)	2.38 (4)	3.195 (9)	178 (11)
N21—H21···Cl3^i^	0.81 (3)	2.45 (5)	3.229 (8)	161 (9)
N31—H31···Cl3	0.82 (3)	2.55 (6)	3.238 (8)	143 (8)
N41—H41···Cl4	0.81 (3)	2.43 (4)	3.234 (8)	172 (10)
N51—H51···Cl4^ii^	0.81 (3)	2.42 (4)	3.209 (9)	165 (10)
N61—H61···Cl4	0.81 (3)	2.50 (6)	3.237 (9)	151 (9)
C1—H1*B*···Cl1	0.99	2.66	3.638 (11)	169
C1—H1*A*···Cl4	0.99	2.51	3.432 (11)	155
C2—H2*A*···Cl2	0.99	2.75	3.710 (11)	162
C2—H2*B*···Cl3^iii^	0.99	2.59	3.431 (10)	143
C36—H36*B*···Au1^i^	0.99	2.87	3.770 (9)	152
C16—H16*A*···Au2	0.99	2.71	3.562 (10)	144
C66—H66*A*···Au3^ii^	0.99	2.91	3.778 (9)	147
C46—H46*B*···Au4	0.99	2.75	3.579 (11)	142
C34—H34···Cl1^iv^	1.00	2.86	3.801 (13)	156
C64—H64···Cl2^v^	1.00	2.81	3.755 (14)	158
C36—H36*A*···Cl3^i^	0.99	2.88	3.571 (9)	127
C42—H42*B*···Cl3^i^	0.99	2.95	3.828 (11)	148
C37—H37*A*···Cl5	0.98	2.90	3.701 (12)	140
C37—H37*B*···Cl6^iv^	0.98	2.94	3.642 (12)	130
C47—H47*C*···Cl6^iv^	0.98	2.77	3.595 (11)	142
C67—H67*C*···Cl7	0.98	2.95	3.770 (12)	142
C15—H15*B*···Cl8^vi^	0.99	2.89	3.709 (11)	141
C17—H17*B*···Cl8^vi^	0.98	2.80	3.637 (11)	143
C55—H55*B*···Cl8	0.99	2.94	3.872 (10)	158

Symmetry codes: (i) −*x*, −*y*+1, −*z*; (ii) −*x*+1, −*y*+1, −*z*+1; (iii) *x*+1, −*y*+1/2, *z*+1/2; (iv) *x*, −*y*+1/2, *z*−1/2; (v) *x*, −*y*+1/2, *z*+1/2; (vi) *x*−1, *y*, *z*.

### Bis(2-methylpiperidine-κN)gold(I) chloride (5) Crystal data

**Table:**

[Au(C_6_H_13_N)_2_]Cl	*F*(000) = 1664
*M**_r_* = 430.76	*D*_x_ = 1.896 Mg m^−^^3^
Monoclinic, *C*2/*c*	Mo *K*α radiation, λ = 0.71073 Å
*a* = 17.6978 (7) Å	Cell parameters from 10304 reflections
*b* = 11.2748 (5) Å	θ = 2.2–30.9°
*c* = 16.5620 (6) Å	µ = 9.90 mm^−^^1^
β = 114.013 (5)°	*T* = 100 K
*V* = 3018.8 (2) Å^3^	Prism, colourless
*Z* = 8	0.2 × 0.2 × 0.1 mm

### Bis(2-methylpiperidine-κN)gold(I) chloride (5) Data collection

**Table:**

Oxford Diffraction Xcalibur, Eos diffractometer	4576 independent reflections
Radiation source: Enhance (Mo) X-ray Source	3012 reflections with *I* > 2σ(*I*)
Detector resolution: 16.1419 pixels mm^-1^	*R*_int_ = 0.051
ω scan	θ_max_ = 31.0°, θ_min_ = 2.2°
Absorption correction: multi-scan (CrysAlisPro; Rigaku OD, 2020)	*h* = −24→24
*T*_min_ = 0.472, *T*_max_ = 1.000	*k* = −16→16
56753 measured reflections	*l* = −23→23

### Bis(2-methylpiperidine-κN)gold(I) chloride (5) Refinement

**Table:**

Refinement on *F*^2^	Primary atom site location: structure-invariant direct methods
Least-squares matrix: full	Secondary atom site location: difference Fourier map
*R*[*F*^2^ > 2σ(*F*^2^)] = 0.029	Hydrogen site location: mixed
*wR*(*F*^2^) = 0.049	H atoms treated by a mixture of independent and constrained refinement
*S* = 1.09	*w* = 1/[σ^2^(*F*_o_^2^) + (0.0126*P*)^2^ + 14.3004*P*] where *P* = (*F*_o_^2^ + 2*F*_c_^2^)/3
4576 reflections	(Δ/σ)_max_ = 0.001
156 parameters	Δρ_max_ = 2.19 e Å^−^^3^
1 restraint	Δρ_min_ = −0.93 e Å^−^^3^

### Bis(2-methylpiperidine-κN)gold(I) chloride (5) Special details

**Table:**

Geometry. Additional structural data: H bonding angle at Cl1: 75.38 ( 1.57) H01 - Cl1 - H02 Torsion angles: -51.28 ( 0.54) C12 - N11 - N11_$1 - C12_$1 74.61 ( 0.32) C12 - N11 - N11_$1 - C16_$1 74.61 ( 0.32) C16 - N11 - N11_$1 - C12_$1 -159.50 ( 0.50) C16 - N11 - N11_$1 - C16_$1 -15.23 ( 0.57) C22 - N21 - N21_$1 - C22_$1 112.02 ( 0.33) C22 - N21 - N21_$1 - C26_$1 112.02 ( 0.33) C26 - N21 - N21_$1 - C22_$1 -120.74 ( 0.55) C26 - N21 - N21_$1 - C26_$1 Symmetry operator: $1 1-x, y, 0.5-z

### Bis(2-methylpiperidine-κN)gold(I) chloride (5) Fractional atomic coordinates and isotropic or equivalent isotropic displacement parameters (Å^2^)

**Table:**

	*x*	*y*	*z*	*U*_iso_*/*U*_eq_	
Au1	0.500000	0.65143 (2)	0.250000	0.01876 (6)	
Au2	0.500000	0.35117 (2)	0.250000	0.02050 (6)	
Cl1	0.67903 (7)	0.47503 (9)	0.49927 (7)	0.0351 (3)	
N11	0.5458 (2)	0.6563 (3)	0.3855 (2)	0.0230 (7)	
H01	0.580 (2)	0.605 (3)	0.404 (3)	0.008 (10)*	
C12	0.5930 (3)	0.7669 (4)	0.4251 (3)	0.0304 (10)	
H12	0.619005	0.756294	0.490706	0.037*	
C13	0.5343 (4)	0.8710 (4)	0.4048 (3)	0.0409 (12)	
H13A	0.511008	0.886761	0.340446	0.049*	
H13B	0.565607	0.942291	0.435336	0.049*	
C14	0.4640 (4)	0.8502 (5)	0.4332 (4)	0.0489 (14)	
H14A	0.486069	0.848206	0.498521	0.059*	
H14B	0.424028	0.916594	0.412179	0.059*	
C15	0.4203 (3)	0.7355 (5)	0.3964 (3)	0.0421 (12)	
H15A	0.378272	0.720215	0.420272	0.050*	
H15B	0.391556	0.741606	0.331276	0.050*	
C16	0.4805 (3)	0.6345 (4)	0.4200 (3)	0.0321 (10)	
H16A	0.450750	0.560046	0.394545	0.039*	
H16B	0.507020	0.625443	0.485098	0.039*	
C17	0.6612 (3)	0.7837 (5)	0.3944 (3)	0.0410 (13)	
H17A	0.692056	0.709351	0.401775	0.062*	
H17B	0.698704	0.846202	0.429470	0.062*	
H17C	0.637539	0.806470	0.331875	0.062*	
N21	0.6268 (2)	0.3476 (3)	0.3152 (2)	0.0254 (7)	
H02	0.635 (3)	0.385 (4)	0.359 (3)	0.038 (15)*	
C22	0.6615 (3)	0.2274 (4)	0.3441 (3)	0.0323 (10)	
H22	0.722096	0.236541	0.381045	0.039*	
C23	0.6517 (3)	0.1515 (4)	0.2636 (3)	0.0385 (11)	
H23A	0.679751	0.074390	0.283897	0.046*	
H23B	0.592258	0.135776	0.228271	0.046*	
C24	0.6881 (3)	0.2127 (5)	0.2056 (3)	0.0467 (14)	
H24A	0.749179	0.213737	0.236952	0.056*	
H24B	0.673745	0.166454	0.150513	0.056*	
C25	0.6573 (3)	0.3380 (5)	0.1823 (3)	0.0428 (12)	
H25A	0.597619	0.336758	0.143016	0.051*	
H25B	0.687020	0.376581	0.150080	0.051*	
C26	0.6706 (3)	0.4085 (5)	0.2647 (3)	0.0357 (11)	
H26A	0.648562	0.489794	0.248173	0.043*	
H26B	0.730526	0.414383	0.302433	0.043*	
C27	0.6239 (3)	0.1748 (5)	0.4001 (3)	0.0410 (12)	
H27A	0.625069	0.232562	0.444791	0.062*	
H27B	0.655037	0.103917	0.429337	0.062*	
H27C	0.566468	0.152896	0.363420	0.062*	

### Bis(2-methylpiperidine-κN)gold(I) chloride (5) Atomic displacement parameters (Å^2^)

**Table:**

	*U*^11^	*U*^22^	*U*^33^	*U*^12^	*U*^13^	*U*^23^
Au1	0.01855 (13)	0.01891 (12)	0.01442 (13)	0.000	0.00220 (10)	0.000
Au2	0.01296 (12)	0.02646 (13)	0.01988 (14)	0.000	0.00442 (11)	0.000
Cl1	0.0340 (5)	0.0342 (7)	0.0243 (5)	0.0089 (4)	−0.0014 (4)	−0.0024 (4)
N11	0.0233 (17)	0.0216 (17)	0.0199 (16)	0.0011 (15)	0.0044 (14)	0.0024 (15)
C12	0.036 (2)	0.033 (2)	0.019 (2)	−0.012 (2)	0.0077 (18)	−0.0035 (18)
C13	0.069 (4)	0.022 (2)	0.038 (3)	−0.004 (2)	0.028 (3)	−0.0012 (19)
C14	0.062 (4)	0.049 (3)	0.047 (3)	0.011 (3)	0.034 (3)	0.003 (3)
C15	0.037 (3)	0.067 (4)	0.025 (2)	0.005 (3)	0.016 (2)	0.003 (2)
C16	0.032 (2)	0.041 (3)	0.021 (2)	−0.012 (2)	0.0074 (18)	0.0022 (19)
C17	0.032 (3)	0.064 (3)	0.026 (3)	−0.021 (2)	0.011 (2)	−0.011 (2)
N21	0.0184 (16)	0.034 (2)	0.0215 (17)	−0.0005 (16)	0.0061 (14)	−0.0041 (17)
C22	0.026 (2)	0.038 (3)	0.030 (2)	0.008 (2)	0.008 (2)	0.005 (2)
C23	0.034 (3)	0.035 (3)	0.040 (3)	0.004 (2)	0.009 (2)	0.000 (2)
C24	0.040 (3)	0.072 (4)	0.029 (3)	0.006 (3)	0.015 (2)	−0.002 (3)
C25	0.033 (3)	0.066 (4)	0.033 (3)	0.004 (3)	0.017 (2)	0.005 (3)
C26	0.021 (2)	0.054 (3)	0.030 (2)	−0.004 (2)	0.0096 (19)	0.001 (2)
C27	0.034 (3)	0.045 (3)	0.043 (3)	0.007 (2)	0.014 (2)	0.012 (2)

### Bis(2-methylpiperidine-κN)gold(I) chloride (5) Geometric parameters (Å, º)

**Table:**

Au1—N11^i^	2.053 (3)	C14—H14A	0.9900
Au1—N11	2.053 (3)	C14—H14B	0.9900
Au1—Au2	3.3854 (3)	C15—H15A	0.9900
Au2—N21	2.057 (3)	C15—H15B	0.9900
Au2—N21^i^	2.057 (3)	C16—H16A	0.9900
N11—C12	1.496 (5)	C16—H16B	0.9900
N11—C16	1.503 (6)	C17—H17A	0.9800
C12—C17	1.501 (6)	C17—H17B	0.9800
C12—C13	1.512 (7)	C17—H17C	0.9800
C13—C14	1.518 (7)	N21—H02	0.80 (3)
C14—C15	1.503 (8)	C22—H22	1.0000
C15—C16	1.498 (7)	C23—H23A	0.9900
N21—C22	1.485 (6)	C23—H23B	0.9900
N21—C26	1.517 (6)	C24—H24A	0.9900
C22—C27	1.469 (7)	C24—H24B	0.9900
C22—C23	1.534 (7)	C25—H25A	0.9900
C23—C24	1.521 (7)	C25—H25B	0.9900
C24—C25	1.507 (7)	C26—H26A	0.9900
C25—C26	1.512 (7)	C26—H26B	0.9900
N11—H01	0.80 (3)	C27—H27A	0.9800
C12—H12	1.0000	C27—H27B	0.9800
C13—H13A	0.9900	C27—H27C	0.9800
C13—H13B	0.9900		
			
N11^i^—Au1—N11	176.9 (2)	C14—C15—H15B	109.5
N11^i^—Au1—Au2	91.54 (10)	H15A—C15—H15B	108.1
N11—Au1—Au2	91.54 (10)	C15—C16—H16A	109.5
N21—Au2—N21^i^	177.8 (2)	N11—C16—H16A	109.5
N21—Au2—Au1	91.11 (11)	C15—C16—H16B	109.5
N21^i^—Au2—Au1	91.11 (11)	N11—C16—H16B	109.5
C12—N11—C16	110.4 (3)	H16A—C16—H16B	108.1
C12—N11—Au1	113.8 (3)	C12—C17—H17A	109.5
C16—N11—Au1	112.9 (3)	C12—C17—H17B	109.5
N11—C12—C17	109.9 (4)	H17A—C17—H17B	109.5
N11—C12—C13	109.8 (4)	C12—C17—H17C	109.5
C17—C12—C13	113.7 (4)	H17A—C17—H17C	109.5
C12—C13—C14	112.8 (4)	H17B—C17—H17C	109.5
C15—C14—C13	111.0 (4)	C22—N21—H02	107 (4)
C16—C15—C14	110.8 (4)	C26—N21—H02	109 (4)
C15—C16—N11	110.5 (4)	Au2—N21—H02	103 (4)
C22—N21—C26	110.6 (4)	C27—C22—H22	107.7
C22—N21—Au2	114.0 (3)	N21—C22—H22	107.7
C26—N21—Au2	113.3 (3)	C23—C22—H22	107.7
C27—C22—N21	109.7 (4)	C24—C23—H23A	109.3
C27—C22—C23	113.8 (4)	C22—C23—H23A	109.3
N21—C22—C23	110.1 (4)	C24—C23—H23B	109.3
C24—C23—C22	111.6 (4)	C22—C23—H23B	109.3
C25—C24—C23	112.6 (4)	H23A—C23—H23B	108.0
C24—C25—C26	110.8 (4)	C25—C24—H24A	109.1
C25—C26—N21	109.4 (4)	C23—C24—H24A	109.1
C12—N11—H01	104 (3)	C25—C24—H24B	109.1
C16—N11—H01	108 (3)	C23—C24—H24B	109.1
Au1—N11—H01	107 (3)	H24A—C24—H24B	107.8
N11—C12—H12	107.8	C24—C25—H25A	109.5
C17—C12—H12	107.8	C26—C25—H25A	109.5
C13—C12—H12	107.8	C24—C25—H25B	109.5
C12—C13—H13A	109.0	C26—C25—H25B	109.5
C14—C13—H13A	109.0	H25A—C25—H25B	108.1
C12—C13—H13B	109.0	C25—C26—H26A	109.8
C14—C13—H13B	109.0	N21—C26—H26A	109.8
H13A—C13—H13B	107.8	C25—C26—H26B	109.8
C15—C14—H14A	109.4	N21—C26—H26B	109.8
C13—C14—H14A	109.4	H26A—C26—H26B	108.2
C15—C14—H14B	109.4	C22—C27—H27A	109.5
C13—C14—H14B	109.4	C22—C27—H27B	109.5
H14A—C14—H14B	108.0	H27A—C27—H27B	109.5
C16—C15—H15A	109.5	C22—C27—H27C	109.5
C14—C15—H15A	109.5	H27A—C27—H27C	109.5
C16—C15—H15B	109.5	H27B—C27—H27C	109.5
			
N11—Au1—Au2—N21^i^	115.79 (14)	C13—C14—C15—C16	53.9 (6)
N11^i^—Au1—Au2—N21^i^	−64.21 (14)	C14—C15—C16—N11	−58.4 (5)
N11—Au1—Au2—N21	−64.21 (14)	C12—N11—C16—C15	60.7 (5)
N11^i^—Au1—Au2—N21	115.79 (14)	Au1—N11—C16—C15	−67.9 (4)
Au2—Au1—N11—C12	154.1 (3)	C26—N21—C22—C27	174.5 (4)
Au2—Au1—N11—C16	−79.1 (3)	Au2—N21—C22—C27	−56.4 (5)
Au1—Au2—N21—C22	172.3 (3)	C26—N21—C22—C23	−59.5 (5)
Au1—Au2—N21—C26	−60.0 (3)	Au2—N21—C22—C23	69.6 (4)
C16—N11—C12—C17	176.6 (4)	C27—C22—C23—C24	177.3 (4)
Au1—N11—C12—C17	−55.3 (4)	N21—C22—C23—C24	53.7 (5)
C16—N11—C12—C13	−57.7 (4)	C22—C23—C24—C25	−50.9 (6)
Au1—N11—C12—C13	70.4 (4)	C23—C24—C25—C26	53.3 (6)
N11—C12—C13—C14	54.2 (5)	C24—C25—C26—N21	−57.8 (5)
C17—C12—C13—C14	177.8 (4)	C22—N21—C26—C25	61.9 (5)
C12—C13—C14—C15	−52.5 (6)	Au2—N21—C26—C25	−67.5 (4)

Symmetry code: (i) −*x*+1, *y*, −*z*+1/2.

### Bis(2-methylpiperidine-κN)gold(I) chloride (5) Hydrogen-bond geometry (Å, º)

**Table:**

*D*—H···*A*	*D*—H	H···*A*	*D*···*A*	*D*—H···*A*
N11—H01···Cl1	0.80 (3)	2.34 (3)	3.110 (4)	162 (4)
N21—H02···Cl1	0.80 (3)	2.36 (3)	3.149 (4)	170 (5)
C27—H27*A*···Cl1	0.98	2.92	3.715 (6)	140
C17—H17*B*···Cl1^ii^	0.98	2.84	3.800 (5)	167
C25—H25*B*···Cl1^iii^	0.99	2.96	3.839 (5)	148
C27—H27*B*···Cl1^iv^	0.98	2.83	3.615 (5)	138
C13—H13*A*···Au1	0.99	3.01	3.436 (5)	107
C15—H15*B*···Au1	0.99	2.94	3.398 (5)	109
C17—H17*C*···Au1	0.98	2.85	3.245 (5)	105
C23—H23*B*···Au2	0.99	3.03	3.440 (5)	106
C25—H25*A*···Au2	0.99	2.94	3.401 (5)	110
C27—H27*C*···Au2	0.98	2.85	3.241 (5)	105

Symmetry codes: (ii) −*x*+3/2, −*y*+3/2, −*z*+1; (iii) *x*, −*y*+1, *z*−1/2; (iv) −*x*+3/2, −*y*+1/2, −*z*+1.
